# A Systematic Review and Meta-Analysis of the Efficacy and Safety of Xinbao Pill in Chronic Heart Failure

**DOI:** 10.3389/fphar.2022.846867

**Published:** 2022-03-02

**Authors:** Yuanping Wang, Yuntao Liu, Zhongqiu Liu, Yuanyuan Cheng, Dawei Wang

**Affiliations:** ^1^ Shunde Hospital of Guangzhou University of Chinese Medicine, Guangzhou University of Chinese Medicine, Guangzhou, China; ^2^ International Institute for Translational Chinese Medicine, Guangzhou University of Chinese Medicine, Guangzhou, China; ^3^ Guangdong Provincial Hospital of Chinese Medicine, The Second Affiliated Hospital, Guangzhou University of Chinese Medicine, Guangzhou, China

**Keywords:** chronic heart failure, Xinbao pill, meta-analysis, systematic review, traditional Chinese medicine (2.592)

## Abstract

**Objective:** This study aimed to clarify the efficacy and safety of Xinbao pill (XBP) as an adjunctive treatment for chronic heart failure (CHF).

**Methods:** Randomized controlled trials (RCTs) on the efficacy and safety of XBP in the treatment of CHF were searched from the six databases. The risk of bias assessment tool recommended by Cochrane Handbook 5.1 were used to assess the methodological quality of the included studies. RevMan 5.3 software was used for meta-analysis. The subgroup and sensitivity analyses were also performed. The grading recommendations assessment, development, and evaluation (GRADE) technique were used to assess the evidence’s certainty.

**Results:** Nine RCTs with a total of 882 patients were identified in this study. The meta-analysis demonstrated that XBP as adjunctive therapy was superior to conventional medicine alone for the treatment of CHF in improving the left ventricular ejection fraction (LVEF; MD = 5.34; 95% CI 4.68 to 5.99; *p* < 0.001), the total effective rate (RR = 1.21; 95% CI, 1.14 to 1.29; *p* < 0.001), the cardiac output (MD = 0.56; 95% CI 0.42 to 0.70; *p* < 0.001), the stroke volume (MD = 3.42; 95% CI 2.03 to 4.81; *p* < 0.001) and the 6-min walking distance (6-MWD; MD = 31.95; 95% CI 21.83 to 42.06; *p* < 0.001), meanwhile reducing the left ventricular end-diastolic diameter (LVEDD; MD = −3.22; 95% CI −4.03 to −2.42; *p* < 0.001) and left ventricular end-systolic dimension (LVESD; MD = −2.93; 95% CI −3.80 to −2.06; *p* < 0.001). Regarding safety, a total of 2.4% (11/456) adverse reactions occurred in the XBP groups while 3.9% (18/456) in the control group. The outcomes’ evidentiary quality ranged from “very low” to “moderate”.

**Conclusion:** This study indicated that XBP as adjunctive therapy combined with conventional medicine seemed to be safe and more effective than conventional medicine alone in treating CHF. However, due to the poor methodological quality of the included RCTs, further well-designed RCTs are required to confirm the efficacy and safety of XBP.

## 1 Introduction

Chronic heart failure (CHF) is a significant and rising global public health issue affecting approximately 64.3 million individuals worldwide (Disease and Prevalence, 2018; Groenewegen et al., 2020). The occurrence of recognized heart failure is believed to be between 1 and 2% of the overall adult population in developed countries (Townsend et al., 2021). It was responsible for an estimated $31 billion (£22.5 billion) in health spending in 2012, accounting for more than 10% of total health spending in the United States for cardiovascular illnesses (Writing Group et al., 2016). Unfortunately, forecasts show that between 2012 and 2030, total costs will rise by 127% (Writing Group et al., 2016). For decades, many types of drugs have been clinically applied for the treatment of CHF, including β-blockers, diuretics, and angiotensin-converting enzyme inhibitors (ACEI), which can more or less relieve the symptoms of CHF (Yancy et al., 2017). However, the available drug treatment options for CHF still do not meet current medical needs, and the 5 years survival rate of patients is only 56.7% (Jones et al., 2019). Although non-pharmacological treatments such as heart transplantation, coronary artery bypass graft surgery, and percutaneous transluminal angioplasty have been used in the treatment of CHF, a significant number of CHF patients still have no access to effective treatments (Stehlik et al., 2018). Therefore, exploring other potentially effective interventions for treating CHF is essential. Many studies have demonstrated that traditional Chinese medicine has a substantial effect on treating CHF in recent years (Hao et al., 2017).

Xinbao pill (XBP) is a Chinese medicine compound prescription composed of *Moschus* (the dried preputial secretion of *Moschus berezovskii, M. sifanicus or M. moschiferus*)*, Panax quinquefolius L.* (Araliaceae), *Cinnamomum verum J. Presl* (*Lauraceae*), *Datura metel L.* (*Solanaceae*), *Aconitum carmichaeli Debeaux* (*Ranunculaceae*), *Panax notoginseng (Burkill) F.H.Chen* (Araliaceae), *Bufonis Venenum* (the dry secretion of *Bufo bufo gargarizans Cantor or Bufo melanostictus Schneider*), *Cervi Cornu Pantotrichum* (the unossitized, densely hairy young horn of a buck by C*ervus Nippon Temminck or Cervus elaphus Linnaeus*), and *Borneolum Syntheticum.* XBP is extensively prescribed for the adjunct management of CHF in China owing to its multiple pharmacological effects on the cardiocerebrovascular system *in vitro* and *in vivo*. It has been shown that XBP could relieve the H2O2-induced H9c2 myocardial cells injury and mitochondrial dysfunction, reduce the oxidative stress level, and adjust the energy metabolism (Li, 2020). In addition, XBP can inhibit cardiac hypertrophy and improve cardiac function in rats with CHF by inhibiting the phosphorylation activation of the PI3K/Akt signal and the phosphorylation of GSK3β (He et al., 2020). Several research trials have focused on the clinical efficacy and safety of XBP as an additional treatment for CHF due to its outstanding efficacy. However, there were no relevant reviews summarizing the efficacy and safety of XBP in the treatment of CHF in terms of quality of methodological and evidence.

We performed a systematic review and meta-analysis based on the available evidence to critically examine the effectiveness and safety of XBP in clinical practice. This study aims to answer two clinical questions for XBP: 1) whether XBP as an adjunct treatment combined with conventional medicine was more effective than conventional medicine alone; 2) whether XBP as an adjunct treatment was safe when used in combination with conventional medicine.

## 2 Materials and Methods

The Preferred Reporting Items for Systematic Reviews and Meta-Analyses (PRISMA) (Page et al., 2021) standards were followed in this study (Supplementary File S1), and the protocol was submitted in PROSPERO (No. CRD 42021236276).

### 2.1 Database and Searching Techniques

From the start until December 2021, a total of six databases, including VIP information resource integration service platform (cqvip), Wanfang Data Knowledge Service Platform, China National Knowledge Infrastructure (CNKI), Cochrane Central Register of Controlled Trial (CENTRAL), embase, and PubMed were searched without regard to language or publishing status. We adopted the search strategy of combining subject words and free words. Additionally, we examined the Chinese Clinical Trials Registry (CHiCTR) and ClinicalTrials.gov for research progress. Moreover, we scanned the reference lists of reviews and meta-analyses. Supplementary File S2 provides a detailed search strategy and search results for the bibliographic databases.

### 2.2 Inclusion and Exclusion Criteria

#### 2.2.1 Type of Studies

Regardless of blinding or publication type, all semi-randomized controlled or randomized controlled trials (RCTs) studies testing the efficacy and safety of XBP for the treatment of CHF were included.

#### 2.2.2 Types of Participants

Adults (age ≥18 years) having a confirmed diagnosis of CHF were included in the study. In an ideal world, diagnostic criteria would be published in articles. Specific diagnostic criteria can refer to “2007 or 2014 Guidelines for the Diagnosis and Treatment of Heart Failure in Chin” or “2016 ESC Guidelines for the diagnosis and treatment of acute and chronic heart failure” (Ponikowski et al., 2016). CHF patients with other diseases (such as coronary heart disease, hypertension, sinus bradycardia, etc.,) were included.

#### 2.2.3 Type of Interventions

Patients treated with conventional medicine, including β-blockers, diuretics, ACEI, angiotensin II receptor blockers, etc., were classified in the control group. In comparison, the intervention group was treated with XBP on the basis of the control group, regardless of the dose, duration, or frequency of administration of XBP. If co-interventions are administered in the intervention group, they should be identical in the control group as well.

#### 2.2.4 Type of Outcome Measures

##### 2.2.4.1 Primary Outcome

➢ Left ventricular ejection fraction (LVEF)
➢ Total effective rate: the signs of evaluation are then compared to the New York Heart Association’s functional classification. It is considered effective when clinical symptoms and signs are reduced, and cardiac function is improved by at least one grade.

##### 2.2.4.2 Secondary Outcomes

➢ Left ventricular end-diastolic dimension (LVEDD)
➢ Left ventricular end-systolic diameter (LVESD)
➢ Cardiac output
➢ Stroke volume
➢ Six-minutes walking distance (6-MWD)

#### 2.2.5 Safety Outcome

Adverse events.

#### 2.2.6 Exclusion Criteria

Articles were eliminated if they matched the following conditions: 1) repeated publication: 2) non-clinical study, fundamental research, review papers, case reports and conceptual discussion. or 3) outcomes data for meta-analysis was missing.

### 2.3 Data Collection and Analysis

#### 2.3.1 Extraction of Data and Quality Valuation

To minimize duplicates, all records were transferred into reference management software (EndNote X9). Two reviewers independently determined the study’s eligibility based on the inclusion/exclusion guidelines. Irrelevant literature, such as reviews and pharmaceutical trials, was excluded by reviewing titles and abstracts. Before confirming inclusion, the complete texts were read. The reviewers evaluated papers with ambiguous titles or abstracts to consider them for selection. If an author published the same data in multiple studies, the most recent publication or the one with the largest sample size was chosen. A standard form was utilized for data extraction to make data statistics easier. It included the following: 1) research ID, 2) size of the sample, 3) initial characteristics of patients (e.g., gender, age), 4) treatment detail (dose, duration and frequency of administration), 5) criteria for CHF diagnosis, and 6) outcomes and adverse reactions. When necessary, the authors of the original studies were consulted for any confusing or missing material. Any discrepancies were handled by a discussion amongst two reviewers or with another researcher.

#### 2.3.2 Evaluation of Risk of Bias

Two researchers independently carried out the quality evaluation based on the risk of bias assessment tool recommended by Cochrane Handbook 5.1 (Shuster, 2011), which was mainly divided into the following six aspects:

1 Generation of random sequence: if random number table, lottery, coin toss, etc., “low risk of bias” is considered; “High risk of bias” was marked when grouping was generated by clinicians determination, patient wishes, laboratory examination results or the admission date and medical record number of participants. If the study only mentioned “randomness”, it was judged as “unclear".
2 Distribution concealment of randomization scheme: if the information was insufficient to make decision, it was judged as “unclear”, those reported randomization, continuous opaque drug containers or closed envelopes controlled by the distribution center were judged as “low risk of bias”. By the contrast, “High risk of bias” was determined if distribution envelopes or drug containers was conducted without protective measures.
3 Blind subjects, researchers, and outcome evaluators: if there was no sufficient information, it was judged as “unclear".
4 The integrity of outcome data;
5 Selective reporting;
6 Other biases.

#### 2.3.3 Data Synthesis and Analysis

The effect size was pooled using the Review Manager Software tool (RevMan, v.5.3; The Cochrane Collaboration). For continuous data, mean deviation (MD) and 95% confidence intervals (CI) were utilized. Dichotomous data were expressed as the relative risk (RR) with 95% CI. The χ2 test and the inconsistency index statistic (*I* ^2^) were used to assess heterogeneity statistically. A random-effect model was used if there was substantial variance (*I* ^2^ > 50% or *p* < 0.05); otherwise, a fixed-effect model was used. Subgroup analysis and sensitivity analysis were also used to investigate potential sources of heterogeneity.

#### 2.3.4 Analysis of the Subgroup

These predetermined subgroup assumptions were used to conduct subgroup analysis.

1 Administration dose of XBP (≤540 mg/day or >540 mg/day);
2 Treatment duration (>8 weeks or ≤8 weeks);
3 In addition, since there were more studies (≥2) testing the efficacy and safety of irbesartan or trimetazidine combined with XBP as intervention groups, subgroup analysis regarding XBP combined with different conventional drugs (irbesartan, trimetazidine) was performed.

#### 2.3.5 Sensitivity Analysis

When significant heterogeneity was detected among studies, sensitivity analysis was computed to evaluate the source of heterogeneity and evaluate whether the decisions at each step of the meta-analysis were stable and credible. This study conducted sensitivity analysis to observe whether the new effect size results and heterogeneity changed significantly after removing single studies.

#### 2.3.6 Publication Bias

For the reason of insufficient number of included studies, publication bias analysis was unable to performed.

#### 2.3.7 Evidence Confidence

The grading recommendations assessment, development, and evaluation (GRADE) technique were used to assess the evidence’s certainty (Goldet and Howick, 2013) following the instructions of the website (https://www.gradepro.org/). RCT evidence is initially classified as high quality, but it can be downgraded due to risk of bias, inaccuracy, inconsistency, informality, and publication bias. The level of evidence is classified into four categories: “high,” “moderate,” “low,” and “very low."

## 3 Results

### 3.1 Search Results

**FIGURE 1:**
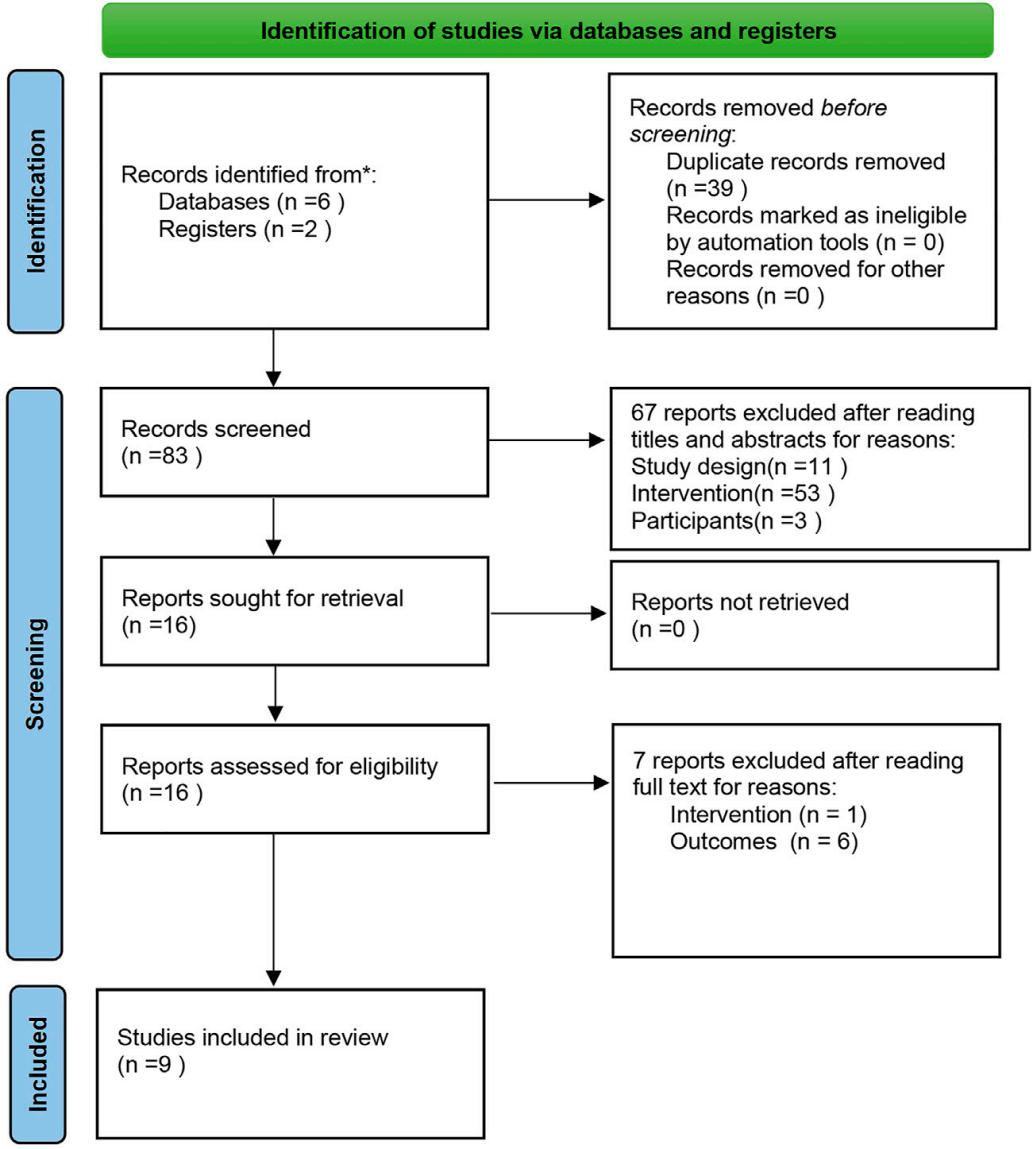
A PRISMA flow diagram of the literature screening and selection process.

A number of 122 relative studies were retrieved, with 83 studies remaining after 39 duplicate studies were eliminated. After reviewing the titles and abstracts, 67 articles were eliminated because they did not match the inclusion requirements. Supplementary File S3 contains a list of studies that were excluded based on the titles and abstracts of studies, and 16 studies were chosen as being potentially relevant. Following a thorough examination of the full text, one of the literature was excluded for the control group was treated conventional medicine combined with betaloc tablets, while the intervention group was treated with conventional medicine combined with XBP, which did not meet the standards of intervention measures. In addition, six literatures were excluded because the outcome indicators were not defined (Supplementary File S4). Finally, this systematic review and meta-analysis contained nine RCTs (Gao et al., 2016; Zhang, 2016; Chen et al., 2018; Li and Lv, 2019; li, 2019; Wang et al., 2019; Xu and Qi, 2019; Zhao et al., 2019; Lu et al., 2020). Figure 1 depicted the study selection procedure.

### 3.2 Included Studies Features

Table 1 and Supplementary File S5 displays the baseline characteristics of all eligible studies. The meta-analysis included nine studies (Gao et al., 2016; Zhang, 2016; Chen et al., 2018; Li and Lv, 2019; li, 2019; Wang et al., 2019; Xu and Qi, 2019; Zhao et al., 2019; Lu et al., 2020) with 882 participants. All studies were conducted in the hospitals of China between 2016 and 2020. The listed studies had sample sizes ranging from 60–120. The treatment duration ranged from 5 days to 12 weeks. In terms of diagnostic criteria, one study (Chen et al., 2018) used 2016 ESC guidelines (Ponikowski et al., 2016) as the diagnostic criteria for CHF, five studies (Zhang, 2016; Li and Lv, 2019; Wang et al., 2019; Xu and Qi, 2019; Zhao et al., 2019) used the diagnostic criteria of the Chinese guidelines (Wu, 2008; Zhang and Zhang, 2014), and three trials (Gao et al., 2016; li, 2019; Lu et al., 2020)did not specify the diagnostic criteria. Two studies (Chen et al., 2018; Li and Lv, 2019) described financing sources as to national or provincial. None of the studies reported follow-up results.

**TABLE1:** Characteristics of included studies.

Study	Country	Study design	Diagnostic criteria	Age (I/C) (years)	No. of patients (I/C)	Female (I/C)	NYHA classification	Co-intervention	Treatment	Comparator	Duration of treatment	Follow-up	Funding
Chen et al. (2018)	China	Rct	2016 ESC guide lines	63.67 ± 8.69/62.92 ± 7.89	50/50	25/24	Ⅱ:19; Ⅲ:19; Ⅳ:12; /Ⅱ:20; Ⅲ:18; Ⅳ:12	CT	XBP,180 mg,tid + irbesartan tablets	Irbesartan tablets	12w	NR	Science and Technology Planning Project of Suzhou City
Gao et al. (2016)	China	Rct	NR	40–73	30/30	NR	Ⅲ ∼ Ⅳ	CT	XBP,120–180 mg,tid	—	8w	NR	NR
Li J (2019)	China	Rct	China’s guideline (2014)	64.86 ± 1.64/64.63 ± 1.47	60/60	27/25	Ⅲ ∼ Ⅳ	CT	XBP:Ⅱ:240 mg,tid; Ⅲ:360 mg,tid + metololol tablets	Metololol tablets	4w	NR	Science and Technology Plan Project of Hebei Provincial Department of Health
Li QJ (2019)	China	Rct	NR	68 ± 8/68 ± 9	40/40	21/13	Ⅱ:12; Ⅲ:22; Ⅳ:6; /Ⅱ:10; Ⅲ:23; Ⅳ:7	CT	XBP,180 mg,tid + Trimetazidine tablets	Trimetazidine tablets	12W	NR	NR
Lu FG (2020)	China	Rct	NR	63.71 ± 6.25/63.85 ± 6.62	60/60	31/18	Ⅱ:18; Ⅲ:28; Ⅳ:14; /Ⅱ:22; Ⅲ:25; Ⅳ:13	CT	XBP,180 mg,tid + irbesartan tablets	Irbesartan tablets	12W	NR	NR
Wang et al. (2019)	China	Rct	China’s guideline (2014)	63.9 ± 6.8/64.7 ± 7.3	48/48	16/19	NR	CT	XBP,360 mg,tid + Milrinone injection	Milrinone injection	5d	NR	NR
Xu and Qi, (2019)	China	Rct	China’s guideline (2014)	53.45 ± 8.11/53.53 ± 8.21	60/60	26/25	Ⅱ:28; Ⅲ:32; /Ⅱ:27; Ⅲ:33	CT	XBP:Ⅱ:240 mg,tid; Ⅲ:360 mg,tid + Carvedilol tablet	Carvedilol tablet	8w	NR	NR
Zhang YY (2016)	China	Rct	China’s guideline (2007)	61.3 ± 7.3/61.3 ± 7.4	50/50	25/24	Ⅱ:18; Ⅲ:25; Ⅳ:7; /Ⅱ:19; Ⅲ:24; Ⅳ:7	CT	XBP,180 mg,tid + Trimetazidine tablets	Trimetazidine tablets	12w	NR	NR
Zhao et al. (2019)	China	Rct	China’s guideline (2007)	83.2 ± 2.1/81.4 ± 1.5	43/43	18/19	Ⅱ:20; Ⅲ:18; Ⅳ:5; /Ⅱ:20; Ⅲ:18; Ⅳ:5	CT	XBP,180 mg,tid + irbesartan tablets	Irbesartan tablets	12w	NR	NR

Abbreviations: I,intervention group; C, control group; XBP, xinbao pill; Rct, randomized controlled trial; CT, conventional therapy; NR, not reported; d,day; w,week; tid, ter in die; NYHA, New York Heart Association’s functional.

### 3.3 Risk of Bias Assessment

Five trials (Chen et al., 2018; li, 2019; Wang et al., 2019; Zhao et al., 2019; Lu et al., 2020) were rated as low risk for using random number tables to generate sequences. One study (Li and Lv, 2019) was grouped according to differences in participants’ medication and was considered high risk, while the other studies (Gao et al., 2016; Zhang, 2016; Xu and Qi, 2019) provided no detailed information about how random sequences are generated. All the included studies published complete data, and no selective outcomes were reported, so the risk of bias was considered “low”. Beyond that, no studies mentioned the information of concealing of allocation, blinding of researchers, participants, and outcome evaluators, result in the risk of bias regarding performance, and detection were considered “unclear”. The risk of other bias was considered “low”, due to no other obvious bias was observed in all RCTs (Table 2).

**TABLE 2:** Risk of bias of included studies.

**Study**	Random sequence generation (selection bias)	Allocation concealment (selection bias)	Blinding of participants and personnel (performance bias)	Blinding of outcome assessment (detection bias)	Incomplete outcome data (attrition bias)	Selective reporting (reporting bias)	Other bias
Chen et al. (2018)	Low risk	Unclear risk	Unclear risk	Unclear risk	Low risk	Low risk	Low risk
Gao et al. (2016)	Unclear risk	Unclear risk	Unclear risk	Unclear risk	Low risk	Low risk	Low risk
Li J (2019)	High risk	Unclear risk	Unclear risk	Unclear risk	Low risk	Low risk	Low risk
Li QJ (2019)	Low risk	Unclear risk	Unclear risk	Unclear risk	Low risk	Low risk	Low risk
Lu FG (2020)	Low risk	Unclear risk	Unclear risk	Unclear risk	Low risk	Low risk	Low risk
Wang et al. (2019)	Low risk	Unclear risk	Unclear risk	Unclear risk	Low risk	Low risk	Low risk
Xu and Qi, (2019)	Unclear risk	Unclear risk	Unclear risk	Unclear risk	Low risk	Low risk	Low risk
Zhang YY (2016)	Unclear risk	Unclear risk	Unclear risk	Unclear risk	Low risk	Low risk	Low risk
Zhao et al. (2019)	Low risk	Unclear risk	Unclear risk	Unclear risk	Low risk	Low risk	Low risk

### 3.4 Meta-Analysis Results

#### 3.4.1 Primary Outcome Measures

**FIGURE 2:**
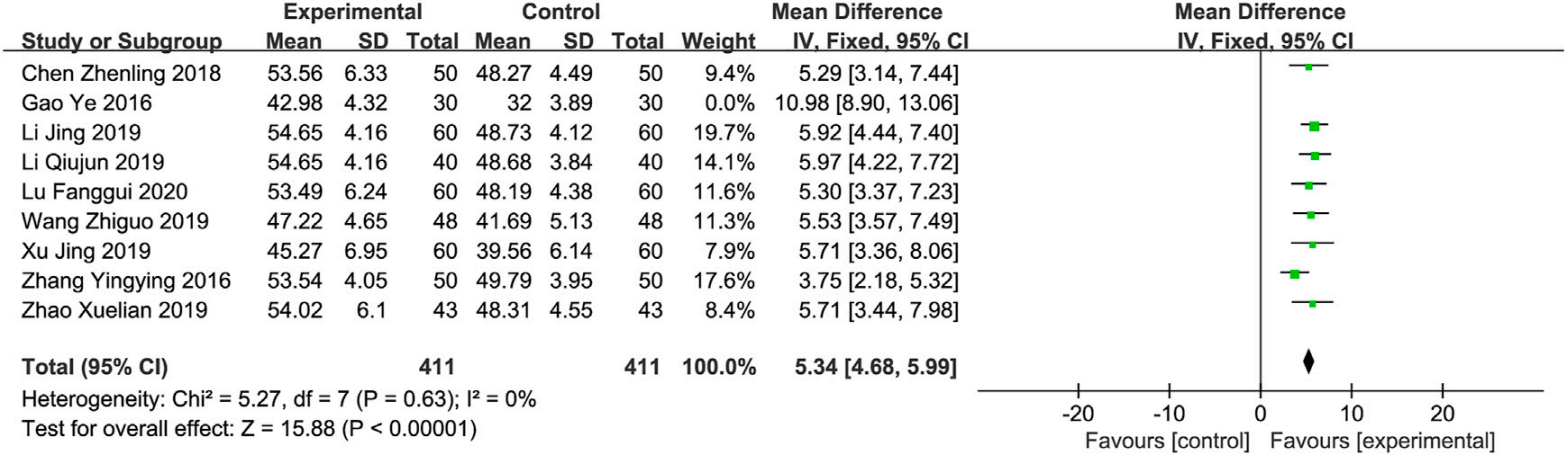
Forest plot of LVEF.

***LVEF*** All the studies reported LVEF (Gao et al., 2016; Zhang, 2016; Chen et al., 2018; Li and Lv, 2019; li, 2019; Wang et al., 2019; Xu and Qi, 2019; Zhao et al., 2019; Lu et al., 2020). The meta-analysis revealed significant heterogeneity in the index level of LVEF (*p* < 0.001 and *I* ^2^ = 74%). Sensitivity analyses were performed by excluding studies one by one. After removing the studies reported by “Gao et al., 2016” (Gao et al., 2016), heterogeneity between studies was significantly reduced (*I* ^2^ = 0%). As shown in Table 1, the sample size of the study “Gao et al., 2016” was the smallest compared to other studies, which might contribute to the heterogeneity. After removing the "“Gao et al., 2016” study, a fixed-effects model was used for meta-analysis. The results showed that on the basis of conventional medicine treatment, combined with XBP, the LVEF value of CHF patients was significantly improved (MD = 5.34; 95% CI (4.68,5.99); *p* < 0.01, Figure 2). Subgroup analyses according to different XBP doses (≤540 mg/day or >540 mg/day), different treatment duration (>8 weeks or ≤8 weeks), and XBP combined with different conventional medicines (irbesartan or trimetazidine) showed no significant difference with these factors (*p* = 0.75, 0.14, and 0.64, respectively) (Supplementary File S6.1–6.3).

**FIGURE 3:**
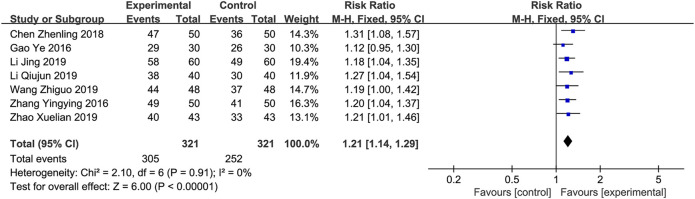
Forest plot of total effective rate.

***Total Effective Rate*** Seven studies (Gao et al., 2016; Zhang, 2016; Chen et al., 2018; Li and Lv, 2019; li, 2019; Wang et al., 2019; Zhao et al., 2019) involving 642 patients reported the total effective rate. Because there was little heterogeneity in this meta-analysis (*p* = 0.91, *I* ^2^ = 0%), a fixed-effects model was used for meta-analysis. As shown in Figure 3, a meta-analysis found that combining XBP and conventional medicine enhance the level of total effective rate compared to conventional medicine alone (RR = 1.21; 95% CI, 1.14 to 1.29; *p* < 0.001). Subgroup analyses according to different XBP doses (≤540 mg/day or >540 mg/day), different treatment duration (>8 weeks or ≤8 weeks), and XBP combined with different conventional medicines (irbesartan or trimetazidine) showed no significant difference with these factors (*p* = 0.65, 0.34, and 0.75, respectively) (Supplementary File S6.4–6.6).

#### 3.4.2 Secondary Outcomes

**FIGURE 4:**
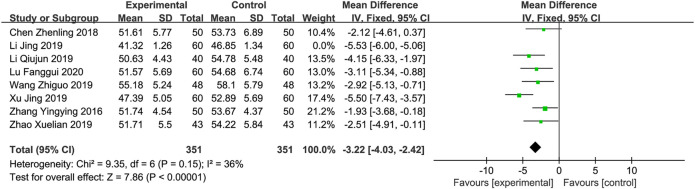
Forest plot of LVEDD.

***LVEDD*** A total of eight RCTs (Zhang, 2016; Chen et al., 2018; Li and Lv, 2019; li, 2019; Wang et al., 2019; Xu and Qi, 2019; Zhao et al., 2019; Lu et al., 2020) involving 822 patients reported the LVEDD. The meta-analysis revealed significant heterogeneity (*p* < 0.001, *I* ^2^ = 79%). Sensitivity analyses were performed by excluding studies one by one. After removing the studies reported by “Li and Lv, 2019” (Li and Lv, 2019), heterogeneity between studies was significantly reduced (*I* ^2^ = 36%). As shown in Table 2, this study failed to perform random sequence generation correctly, and its selection bias was considered to be high risk, which might contribute to the heterogeneity. After removing the “Li and Lv, 2019” study, a fixed-effects model was used for meta-analysis. Meta-analysis revealed that XBP combined with conventional medicine treatment decreases the level of LVEDD, which is better than using conventional medicine alone (MD = -3.22; 95% CI -4.03 to -2.42; *p* < 0.001, Figure 4). Subgroup analyses according to XBP combined with different conventional medicines (irbesartan or trimetazidine) showed no significant difference with this factor (*p* = 0.86). However, subgroup analysis according to different XBP doses (≤540 mg/day or >540 mg/day) or different treatment duration (>8 weeks or ≤8 weeks) showed significant subgroup difference (*p* = 0.01 and 0.01, respectively) and the heterogeneities in these two subgroups were decreased, suggesting that XBP dose and treatment duration may be a potential source of heterogeneity (Supplementary File S6.7–6.9).

**FIGURE 5:**
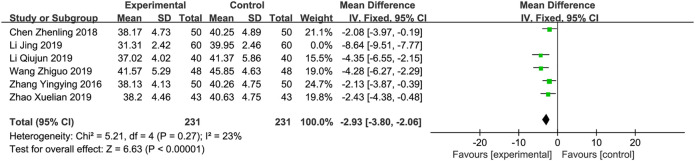
Forest plot of LVESD.

***LVESD*** A total of six trials (Zhang, 2016; Chen et al., 2018; Li and Lv, 2019; li, 2019; Wang et al., 2019; Zhao et al., 2019) involving 582 patients reported the LVESD. The results of a meta-analysis revealed that there was significant heterogeneity (*p* < 0.001, *I* ^2^ = 94%). Sensitivity analyses were performed by excluding studies one by one. After removing the studies reported by “Li and Lv, 2019”, heterogeneity between studies was significantly reduced (*I* ^2^ = 23%). After removing this study, a fixed-effects model was used for meta-analysis. Meta-analysis revealed that XBP combined with conventional medicine treatment reduces the level of LVEDD, which is better than using conventional medicine alone (MD = −2.93; 95% CI −3.80 to −2.06; *p* < 0.001, Figure 5). Subgroup analyses according to XBP combined with different conventional medicines (irbesartan or trimetazidine) showed no significant difference with this factor (*p* = 0.45). However, subgroup analysis according to different XBP doses (≤540 mg/day or >540 mg/day) or different treatment duration (>8 weeks or ≤8 weeks) showed significant subgroup difference (*p* = 0.08 and 0.08, respectively) and the heterogeneities in these two subgroups were decreased, suggesting that XBP dose and treatment duration may be a potential source of heterogeneity (Supplementary File S6.10–6.12).

**FIGURE 6:**
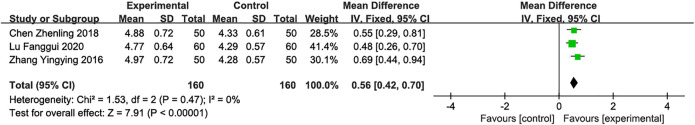
Forest plot of cardiac output.

***Cardiac output*** Three studies (Zhang, 2016; Chen et al., 2018; Lu et al., 2020) involving 320 patients reported the cardiac output. As low heterogeneity was found in this meta-analysis (*p* = 0.47, *I* ^*2*^ = 0%), a fixed-model of effects was conducted for meta-analysis to estimate the MD. A meta-analysis found that combining XBP with conventional medicine therapy can boost cardiac output when relative to conventional medicine alone (MD = 0.56; 95% CI 0.42 to 0.70; *p* < 0.001) (Figure 6). Since few studies reported cardiac output, subgroup analysis could not be performed.

**FIGURE 7:**
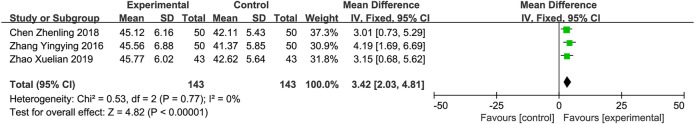
Forest plot of stroke volume.

***Stroke volume*** Three studies (Zhang, 2016; Chen et al., 2018; Zhao et al., 2019) involving 286 patients reported the stroke volume. As low heterogeneity was found in this meta-analysis (*p* = 0.77, *I* ^*2*^ = 0%), a fixed-model of effects was conducted for meta-analysis to estimate the MD. Meta-analysis revealed that the XBP and conventional medicine combined treatment may increase the stroke volume level in comparison to conventional medicine only (MD = 3.42; 95% CI 2.03 to 4.81; *p* < 0.001) (Figure 7). Since few studies reported cardiac output, subgroup analysis could not be performed.

**FIGURE 8:**
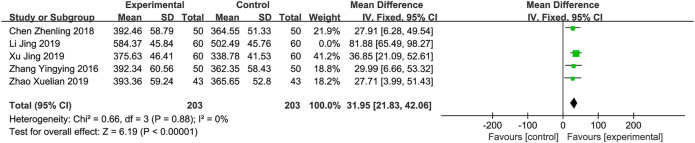
Forest plot of 6-MWD.

***6-MWD*** A total of five studies (Zhang, 2016; Chen et al., 2018; Li and Lv, 2019; Xu and Qi, 2019; Zhao et al., 2019) involving 526 patients reported the 6-MWD. The results of a meta-analysis revealed that there was significant heterogeneity (*p* < 0.001, *I* ^2^ = 85%). Sensitivity analyses were performed by excluding studies one by one. After removing the studies reported by “Li and Lv, 2019”, heterogeneity between studies was significantly reduced (*I* ^2^ = 0%). After removing this study, a fixed-effects model was used for meta-analysis. Meta-analysis indicated that XBP combined with conventional medicine treatment improves the level of 6-MWD, which is better than using conventional medicine alone (MD = 31.95; 95% CI 21.83 to 42.06; *p* < 0.001, Figure 8). Subgroup analyses according to different XBP doses (≤540 mg/day or >540 mg/day), and different treatment duration (>8 weeks or ≤8 weeks) showed no significant difference with these factors (*p* = 0.19, and 0.19, respectively) (Supplementary File S6.13–6.14).

### 3.5 Adverse Events

Adverse events were mentioned in four trials (Li and Lv, 2019; Wang et al., 2019; Xu and Qi, 2019; Lu et al., 2020). In one study (Li and Lv, 2019), no adverse reactions were observed in both groups during treatment. Three of the studies (Wang et al., 2019; Xu and Qi, 2019; Lu et al., 2020) reported a total of 12.4% (11/456) adverse reactions in the XBP group and 3.9% (18/456) adverse reactions in the control group. All of the adverse reactions were modest, and no significant adverse events were observed, detailed information is shown in Table 3.

**TABLE 3:** The incidence rate of adverse effect.

**Type**	Study	The number of adverse effect	
		Intervention group	Control group
Dizziness	Lu FG, (2020); Xu and Qi, (2019)	2	7
Fatigue	Lu FG, (2020); Xu and Qi (2019)	3	5
Chest tightness	Lu FG, (2020)	2	3
Hypotension	Wang et al. (2019)	2	1
Arrhythmias	Wang et al. (2019)	0	1
Dry cough	Wang et al. (2019)	1	0
Disturbed sleep	Xu and Qi, (2019)	0	1
Rash	Xu and Qi, (2019)	1	0
Total event	—	11/456	18/456
Incidence rate	—	2.4%	3.9%

### 3.6 The Quality of the Evidence

The outcomes’ evidentiary quality ranged from “very low” to “moderate” The rationale for the downgrade was the selected studies’ unclear risk of bias, inconsistency of results due to significant heterogeneity, and imprecision of results due to small sample sizes (Supplementary File S7).

## 4 Discussion

### 4.1 Main Results of This Research

The current systematic review compared the efficacy and safety of XBP coupled with conventional medicine to conventional medicine alone in CHF. This study contained nine RCTs (Gao et al., 2016; Zhang, 2016; Chen et al., 2018; Li and Lv, 2019; li, 2019; Wang et al., 2019; Xu and Qi, 2019; Zhao et al., 2019; Lu et al., 2020) and a meta-analysis of 35 outcomes was performed.

The data indicated that there was evidence was graded as very low to moderate quality that XBP in conjunction with conventional medicine played superior advantages in improving LVEF, total efficacy rate, stroke volume, cardiac output, and 6-MWD, as well as decreasing LVEDD and LVESD, compared to that of conventional medicine alone. In addition, to discuss the effects of administration doses, treatment duration, and co-intervention (irbesartan or trimetazidine) of XBP for CHF treatment, we arranged subgroup analyses, which showed that XBP improved LVEF, and reduced LVEDD and LVESD, regardless of medication dose (≤540 mg or >540 mg), treatment duration (≤8 weeks, or >8 weeks) or types of combination medications. Interestingly, the benefit of XBP in CHF persisted regardless of subgroup analysis.

Regarding clinical safety, a total of 2.4% (11/456) adverse reactions occurred in the XBP groups while 3.9% (18/456) in the control group. However, it was insufficient evidence to advocate that combination therapy was safer than conventional medicine since only 4 (44.4%) eligible studies had recorded adverse effects (dizziness, fatigue, chest tightness, hypertension, arrythmias, dry cough, disturbed sleep and rash) (Li and Lv, 2019; Wang et al., 2019; Xu and Qi, 2019; Lu et al., 2020). Additionally, we dated back to the instructions of XBP, and “unclear” was recorded for adverse effects, implying that there was no credible clinical evidence to examine the safety of XBP. As a consequence, the safety of XBP in the treatment of CHF is yet unknown, further clinical trials are required to validate this in the future.

### 4.2 Evidence’s Confidence

GRADE approach was used to assess the confidence of the evidence in this study and the results revealed that the majority of outcomes were graded as very low to moderate quality. It was not difficult to find out that substantial risk of bias, inconsistency among research, and imprecision of the findings took responsibility for the evidence downgrading. As a result, the following adoption of larger RCTs with improved methodological quality is anticipated to have a major impact on the conclusions of this review.

### 4.3 Bias Risk

None of the studies included in this analysis adequately concealed and blinded random allocation, which may result in an overstated effect of treatment. Furthermore, no studies had employed intention-to-treat analysis, which would skew the results in favor of XBP. Taking these points into consideration, we could not rule out that the effect magnitude found in this study may be exaggerated. As a whole, developed on the low-quality methodology, the findings should be evaluated and utilized cautiously.

### 4.4 Between-Studies Heterogeneity

Clinical variation or methodological diversity (study design and risk of bias) or both, across studies, consequently caused statistical heterogeneity (Melsen et al., 2014). In particular, determining the source of heterogeneity among results of studies is interest, the present systematic review addressed clinical heterogeneity by carefully specifying population, interventions, comparators, outcomes and study designs. In addition, we also performed subgroup analysis according to the dose of XBP, the duration of treatment and the combination of different drugs with XBP to minimize the clinical heterogeneity of included studies. However, there were inevitably some clinical heterogeneities. First, our inclusion criteria for the intervention group was the combination of XBP treatment on the basis of the control group’s conventional drug treatment. However, in the original study, the details of the use of conventionally treated drugs were not described in detail. Considering that these included studies were all carried out in county-level public hospitals in China, and the medical level in hospitals varies, we were not confident about whether the specific routine medication for heart failure was standardized and whether the recommendations of clinical guidelines are followed. Moreover patients with clinical CHF generally suffering from multiple diseases, such as hypertension, coronary heart disease, atrial fibrillation and other cardiovascular diseases. Nevertheless the included studies lacked detailed of underlying health conditions which also is an important source of heterogeneity for this unclear and inconsistent clinical characters. For the methodological heterogeneity identified in LVEF, we did sensitivity analyses to reduce the heterogeneity by removing one small sample size study (Gao et al., 2016). Likewise, the significant heterogeneity in the result of LVEDD, LVESD, and 6-MWD were reduced by excluding a high risk of randomization study (Xu and Qi, 2019). We believed that inappropriate study design and unreasonable randomization were also potential sources of heterogeneity.

### 4.5 Publication Bias

We did not examine publication bias in this analysis since there were less than ten studies included. Furthermore, we were unable to detect selectively reporting results since none of the original trials in this analysis presented the information of clinical trial registry or study protocol. For these reasons, we cannot rule out the likelihood of publication bias for the time being.

### 4.6 Clinical Practice Implications and Prospective Research

Due to a dearth of high-quality evidence, this research was unable to draw any definite conclusions about the effects and safety of XBP. As a consequence of the uncertainty efficacy, clinicians are reminded to make more thoughtful decisions when prescribing this medication until further solid clinical studies are available. Additionally, while some trials have reported adverse events, the safety of XBP is still largely unclear, and clinicians and patients should pay close attention to medication.

More high-quality studies are being or on being conducted to examine the efficacy of XBP, and the following points are indicated for special consideration in future research based on the methodological evaluation conducted in this study:

• Use accepted diagnostic criteria or provide references for detailed explanations.
• The estimation of sample size should be more rigorous and scientific.
• The placebo should be considered in study design to exclude the placebo effect of XBP.
• Describe methods for generating randomly assigned sequences.
• Outcome indicators should be selected more extensively, such as N-terminal pro-brain natriuretic peptide, B-type natriuretic peptide and the Minnesota Living with Heart Failure Questionnaire, etc.
• Detailed records of adverse events during the trial process to evaluate the safety of XBP.
• After the clinical trial is over, patients should be followed up to evaluate the long-term efficacy of XBP.
• The geographical and ethnicity of the study needs to be expanded to determine the broad applicability of the XBP.

### 4.7 Limitation of the Present Study

This is the first systematic review to summarize the efficacy and safety of XBP in the treatment of CHF. The assessing the methodological quality of systematic reviews-2 (AMSTAR 2) (Shea et al., 2017) approach was used to assess the methodological quality of this study, and it was determined to be of a high standard (Supplementary File S8). Additionally, we have included comprehensive Supplementary Materials that enable replication and review of this work. Although the adoption of high-quality techniques, major restrictions are unavoidably present. To begin, this study contained a small number of studies, and there is doubt regarding the clinical efficacy of XBP in the treatment of CHF. Second, although we did not set the language restriction for inclusion, the search strategies for potential researches screening were limited to Chinese and English databases and those published in Japanese and Korean were missed, which may inevitably have a certain selective bias. Thirdly, only five studies precisely defined the randomization procedure for the risk of bias evaluation of the included studies, and the risk of bias for the remaining papers was unknown, resulting in some risk of bias in the analysis. Additionally, because the majority of the patients in the included studies were Chinese, the conclusion of this meta-analysis may not apply to other ethnic groups. Finally, the quality of the clinical research included was low to very low, implying that future in-depth trials should include more trustworthy clinical evidence to support the reasonable therapeutic application of XBP.

## 5 Conclusion

According to current evidence, as compared to the use of conventional medicine alone, the combined use of XBP had more advantages in improving the clinical effectiveness of CHF patients, increasing the value of LVEF, stroke volume, cardiac output, 6-MWD, and reducing the value of LVEDD and LVESD, along with good safety outcomes. Though, given the low quality of the included studies, the lack of placebo control, and the substantial variability among eligible trials, we should proceed with caution. Furthermore, the safety of XBP is unknown, more high-quality controlled trials are required in the future to confirm the efficacy and safety of this medicine.

## Data Availability

The original contributions presented in the study are included in the article/Supplementary Material, further inquiries can be directed to the corresponding authors.

## Abbreviations

ACEI, Angiotensin-converting enzyme inhibitors; AMSTAR 2, Assessing the Methodological Quality of Systematic Reviews-2; CHF, chronic heart failure; CI, confidence interval; GRADE, Grading recommendations assessment, development, and evaluation; 6-MWD, six-minutes walking distance; MD, mean difference; LVEF, left ventricular ejection fraction; LVEDD, left ventricular end-diastolic dimension; LVESD, left ventricular end-systolic diameter, RCTs, randomized controlled trials; RR, relative risk; XBP, Xinbao pill.
