# Supplementary material for: A Systematic Review and Meta-Analysis of the Efficacy and Safety of Xinbao Pill in Chronic Heart Failure
Source: Front Pharmacol. 2022 Mar 2;13:846867. doi: 10.3389/fphar.2022.846867 (PMC8924547; doi:10.3389/fphar.2022.846867)
Supplement: Supplementary file 1 [file DataSheet1.DOCX]

**Table of contents**

[Supplementary File S1. PRISMA 2020 checklist 2](#_Toc13637)

[Supplementary File S2. Search strategies for databases. 6](#_Toc16468)

[Supplementary File S3. A list of excluded studies by reading title and abstract. 24](#_Toc22408)

[Supplementary File S4. A list of excluded studies by reading full text. 29](#_Toc27512)

[Supplementary File S5. Summary table of the studies include. 30](#_Toc23279)

[Supplementary File S6. Results of subgroup analysis 38](#_Toc8931)

[Supplementary File S7. Results of meta-analysis and quality of evidence 44](#_Toc32623)

[Supplementary File S8. AMSTAR 2 Checklist 48](#_Toc9787)

# Supplementary File S1. PRISMA 2020 checklist

| **Section and Topic** | **Item #** | **Checklist item** | **Location where item is reported** |
| --- | --- | --- | --- |
| **TITLE** | | |  |
| Title | 1 | Identify the report as a systematic review. | P1 |
| **ABSTRACT** | | |  |
| Abstract | 2 | See the PRISMA 2020 for Abstracts checklist. | P2 |
| **INTRODUCTION** | | |  |
| Rationale | 3 | Describe the rationale for the review in the context of existing knowledge. | P3 |
| Objectives | 4 | Provide an explicit statement of the objective(s) or question(s) the review addresses. | P3 |
| **METHODS** | | |  |
| Eligibility criteria | 5 | Specify the inclusion and exclusion criteria for the review and how studies were grouped for the syntheses. | P4-5 |
| Information sources | 6 | Specify all databases, registers, websites, organisations, reference lists and other sources searched or consulted to identify studies. Specify the date when each source was last searched or consulted. | P4 |
| Search strategy | 7 | Present the full search strategies for all databases, registers and websites, including any filters and limits used. | P4 |
| Selection process | 8 | Specify the methods used to decide whether a study met the inclusion criteria of the review, including how many reviewers screened each record and each report retrieved, whether they worked independently, and if applicable, details of automation tools used in the process. | P5 |
| Data collection process | 9 | Specify the methods used to collect data from reports, including how many reviewers collected data from each report, whether they worked independently, any processes for obtaining or confirming data from study investigators, and if applicable, details of automation tools used in the process. | P5 |
| Data items | 10a | List and define all outcomes for which data were sought. Specify whether all results that were compatible with each outcome domain in each study were sought (e.g. for all measures, time points, analyses), and if not, the methods used to decide which results to collect. | P4-5 |
|  | 10b | List and define all other variables for which data were sought (e.g. participant and intervention characteristics, funding sources). Describe any assumptions made about any missing or unclear information. | P4-5 |
| Study risk of bias assessment | 11 | Specify the methods used to assess risk of bias in the included studies, including details of the tool(s) used, how many reviewers assessed each study and whether they worked independently, and if applicable, details of automation tools used in the process. | P5-6 |
| Effect measures | 12 | Specify for each outcome the effect measure(s) (e.g. risk ratio, mean difference) used in the synthesis or presentation of results. | P6 |
| Synthesis methods | 13a | Describe the processes used to decide which studies were eligible for each synthesis (e.g. tabulating the study intervention characteristics and comparing against the planned groups for each synthesis (item #5)). | P6 |
|  | 13b | Describe any methods required to prepare the data for presentation or synthesis, such as handling of missing summary statistics, or data conversions. | P6 |
|  | 13c | Describe any methods used to tabulate or visually display results of individual studies and syntheses. | P6 |
|  | 13d | Describe any methods used to synthesize results and provide a rationale for the choice(s). If meta-analysis was performed, describe the model(s), method(s) to identify the presence and extent of statistical heterogeneity, and software package(s) used. | P6 |
|  | 13e | Describe any methods used to explore possible causes of heterogeneity among study results (e.g. subgroup analysis, meta-regression). |  |
|  | 13f | Describe any sensitivity analyses conducted to assess robustness of the synthesized results. | P6 |
| Reporting bias assessment | 14 | Describe any methods used to assess risk of bias due to missing results in a synthesis (arising from reporting biases). | P6 |
| Certainty assessment | 15 | Describe any methods used to assess certainty (or confidence) in the body of evidence for an outcome. | P6 |
| **RESULTS** | | |  |
| Study selection | 16a | Describe the results of the search and selection process, from the number of records identified in the search to the number of studies included in the review, ideally using a flow diagram. | P7 |
|  | 16b | Cite studies that might appear to meet the inclusion criteria, but which were excluded, and explain why they were excluded. | P7 |
| Study characteristics | 17 | Cite each included study and present its characteristics. | P7 |
| Risk of bias in studies | 18 | Present assessments of risk of bias for each included study. | P7 |
| Results of individual studies | 19 | For all outcomes, present, for each study: (a) summary statistics for each group (where appropriate) and (b) an effect estimate and its precision (e.g. confidence/credible interval), ideally using structured tables or plots. | P7-9 |
| Results of syntheses | 20a | For each synthesis, briefly summarise the characteristics and risk of bias among contributing studies. | P7-9 |
|  | 20b | Present results of all statistical syntheses conducted. If meta-analysis was done, present for each the summary estimate and its precision (e.g. confidence/credible interval) and measures of statistical heterogeneity. If comparing groups, describe the direction of the effect. | P7-9 |
|  | 20c | Present results of all investigations of possible causes of heterogeneity among study results. | P7-9 |
|  | 20d | Present results of all sensitivity analyses conducted to assess the robustness of the synthesized results. | P7-9 |
| Reporting biases | 21 | Present assessments of risk of bias due to missing results (arising from reporting biases) for each synthesis assessed. | NA |
| Certainty of evidence | 22 | Present assessments of certainty (or confidence) in the body of evidence for each outcome assessed. | P10 |
| **DISCUSSION** | | |  |
| Discussion | 23a | Provide a general interpretation of the results in the context of other evidence. | P10-11 |
|  | 23b | Discuss any limitations of the evidence included in the review. | P12-13 |
|  | 23c | Discuss any limitations of the review processes used. | P12-13 |
|  | 23d | Discuss implications of the results for practice, policy, and future research. | P12 |
| **OTHER INFORMATION** | | |  |
| Registration and protocol | 24a | Provide registration information for the review, including register name and registration number, or state that the review was not registered. | P4 |
|  | 24b | Indicate where the review protocol can be accessed, or state that a protocol was not prepared. | P4 |
|  | 24c | Describe and explain any amendments to information provided at registration or in the protocol. | P4 |
| Support | 25 | Describe sources of financial or non-financial support for the review, and the role of the funders or sponsors in the review. | P13 |
| Competing interests | 26 | Declare any competing interests of review authors. | P13 |
| Availability of data, code and other materials | 27 | Report which of the following are publicly available and where they can be found: template data collection forms; data extracted from included studies; data used for all analyses; analytic code; any other materials used in the review. | P13 |

*From:*  Page MJ, McKenzie JE, Bossuyt PM, Boutron I, Hoffmann TC, Mulrow CD, et al. The PRISMA 2020 statement: an updated guideline for reporting systematic reviews. BMJ 2021;372:n71. doi: 10.1136/bmj.n71

For more information, visit: <http://www.prisma-statement.org/>

# Supplementary File S2. Search strategies for databases.

2.1 PubMed (https://pubmed.ncbi.nlm.nih.gov/advanced/)

**Search date:December 19, 2021 Beijing time**

| Search | Add to builder | | Query | Iterms found | Time |
| --- | --- | --- | --- | --- | --- |
| 5 | (Xinbao wan[Title/Abstract] OR Xinbao pill[Title/Abstract]) AND ((Cardiac Failure[Title/Abstract] OR Heart Decompensation[Title/Abstract] OR Decompensation, Heart[Title/Abstract] OR Heart Failure, Right-Sided[Title/Abstract] OR Heart Failure, Right Sided[Title/Abstract] OR Right-Sided Heart Failure[Title/Abstract] OR Right Sided Heart Failure[Title/Abstract] OR Myocardial Failure[Title/Abstract] OR Congestive Heart Failure[Title/Abstract] OR Heart Failure, Congestive[Title/Abstract] OR Heart Failure, Left-Sided[Title/Abstract] OR Heart Failure, Left Sided[Title/Abstract] OR Left-Sided Heart Failure[Title/Abstract] OR Left Sided Heart Failure[Title/Abstract]) OR (Chronic heart failure[MeSH Terms])) - Schema: all | | (Xinbao wan[Title/Abstract] OR Xinbao pill[Title/Abstract]) AND ((Cardiac Failure[Title/Abstract] OR Heart Decompensation[Title/Abstract] OR Decompensation, Heart[Title/Abstract] OR Heart Failure, Right-Sided[Title/Abstract] OR Heart Failure, Right Sided[Title/Abstract] OR Right-Sided Heart Failure[Title/Abstract] OR Right Sided Heart Failure[Title/Abstract] OR Myocardial Failure[Title/Abstract] OR Congestive Heart Failure[Title/Abstract] OR Heart Failure, Congestive[Title/Abstract] OR Heart Failure, Left-Sided[Title/Abstract] OR Heart Failure, Left Sided[Title/Abstract] OR Left-Sided Heart Failure[Title/Abstract] OR Left Sided Heart Failure[Title/Abstract]) OR (Chronic heart failure[MeSH Terms])) | 0 | 19:30:56 |
| 4 | Xinbao wan[Title/Abstract] OR Xinbao pill[Title/Abstract] | | ("Xinbao"[All Fields] AND "wan"[Title/Abstract]) OR ("Xinbao"[All Fields] AND "pill"[Title/Abstract]) | 2 | 19:28:19 |
| 3 | (Cardiac Failure[Title/Abstract] OR Heart Decompensation[Title/Abstract] OR Decompensation, Heart[Title/Abstract] OR Heart Failure, Right-Sided[Title/Abstract] OR Heart Failure, Right Sided[Title/Abstract] OR Right-Sided Heart Failure[Title/Abstract] OR Right Sided Heart Failure[Title/Abstract] OR Myocardial Failure[Title/Abstract] OR Congestive Heart Failure[Title/Abstract] OR Heart Failure, Congestive[Title/Abstract] OR Heart Failure, Left-Sided[Title/Abstract] OR Heart Failure, Left Sided[Title/Abstract] OR Left-Sided Heart Failure[Title/Abstract] OR Left Sided Heart Failure[Title/Abstract]) OR (Chronic heart failure[MeSH Terms]) | | "cardiac failure"[Title/Abstract] OR "heart decompensation"[Title/Abstract] OR "decompensation heart"[Title/Abstract] OR "heart failure right sided"[Title/Abstract] OR "heart failure right sided"[Title/Abstract] OR "right sided heart failure"[Title/Abstract] OR "right sided heart failure"[Title/Abstract] OR "myocardial failure"[Title/Abstract] OR "congestive heart failure"[Title/Abstract] OR "heart failure congestive"[Title/Abstract] OR "heart failure left sided"[Title/Abstract] OR "heart failure left sided"[Title/Abstract] OR "left sided heart failure"[Title/Abstract] OR "left sided heart failure"[Title/Abstract] OR (("chronic"[All Fields] OR "chronical"[All Fields] OR "chronically"[All Fields] OR "chronicities"[All Fields] OR "chronicity"[All Fields] OR "chronicization"[All Fields] OR "chronics"[All Fields]) AND "heart failure"[MeSH Terms]) | 80,026 | 19:25:25 |
| 2 | Cardiac Failure[Title/Abstract] OR Heart Decompensation[Title/Abstract] OR Decompensation, Heart[Title/Abstract] OR Heart Failure, Right-Sided[Title/Abstract] OR Heart Failure, Right Sided[Title/Abstract] OR Right-Sided Heart Failure[Title/Abstract] OR Right Sided Heart Failure[Title/Abstract] OR Myocardial Failure[Title/Abstract] OR Congestive Heart Failure[Title/Abstract] OR Heart Failure, Congestive[Title/Abstract] OR Heart Failure, Left-Sided[Title/Abstract] OR Heart Failure, Left Sided[Title/Abstract] OR Left-Sided Heart Failure[Title/Abstract] OR Left Sided Heart Failure[Title/Abstract] | | "cardiac failure"[Title/Abstract] OR "heart decompensation"[Title/Abstract] OR "decompensation heart"[Title/Abstract] OR "heart failure right sided"[Title/Abstract] OR "heart failure right sided"[Title/Abstract] OR "right sided heart failure"[Title/Abstract] OR "right sided heart failure"[Title/Abstract] OR "myocardial failure"[Title/Abstract] OR "congestive heart failure"[Title/Abstract] OR "heart failure congestive"[Title/Abstract] OR "heart failure left sided"[Title/Abstract] OR "heart failure left sided"[Title/Abstract] OR "left sided heart failure"[Title/Abstract] OR "left sided heart failure"[Title/Abstract] | 57,510 | 19:24:40 |
| 1 | Chronic heart failure[MeSH Terms] |  | ("chronic"[All Fields] OR "chronical"[All Fields] OR "chronically"[All Fields] OR "chronicities"[All Fields] OR "chronicity"[All Fields] OR "chronicization"[All Fields] OR "chronics"[All Fields]) AND "heart failure"[MeSH Terms] | 28,004 | 19:23:29 |


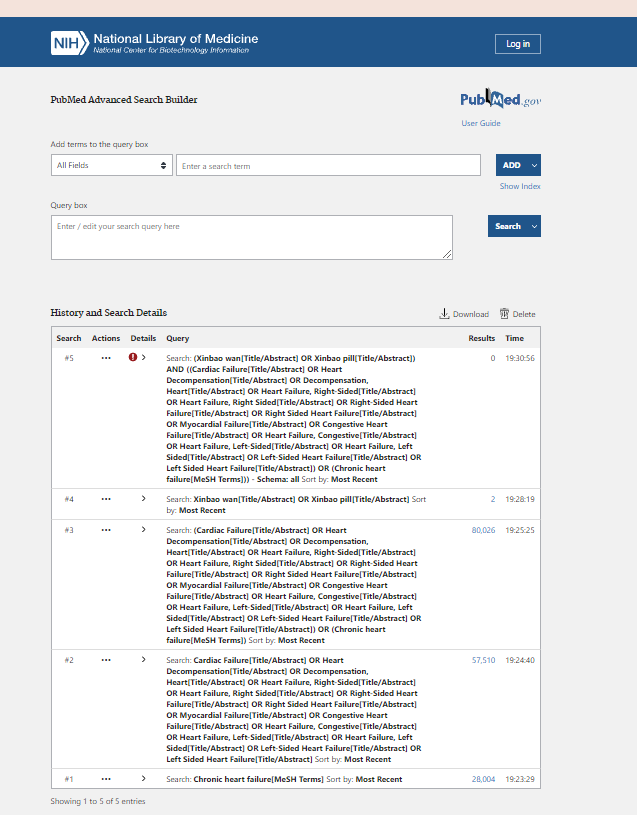


2.2 Embase (https://www.embase.com/)

**Search date:December 19, 2021 Beijing time**

| No. | Query | Results |
| --- | --- | --- |
| #3 | #1 AND #2 | 0 |
| #2 | 'xinbao pill':ab,ti OR 'xinbao wan':ab,ti | 3 |
| #1 | 'chronic heart failure':ab,ti OR 'cardiac failure':ab,ti OR 'heart decompensation':ab,ti OR 'decompensation, heart':ab,ti OR 'heart failure, right-sided':ab,ti OR 'heart failure, right sided':ab,ti OR 'right-sided heart failure':ab,ti OR 'right sided heart failure':ab,ti OR 'myocardial failure':ab,ti OR 'congestive heart failure':ab,ti OR 'heart failure, congestive':ab,ti OR 'heart failure, left-sided':ab,ti OR 'heart failure, left sided':ab,ti OR 'left-sided heart failure':ab,ti OR 'left sided heart failure':ab,ti | 104302 |


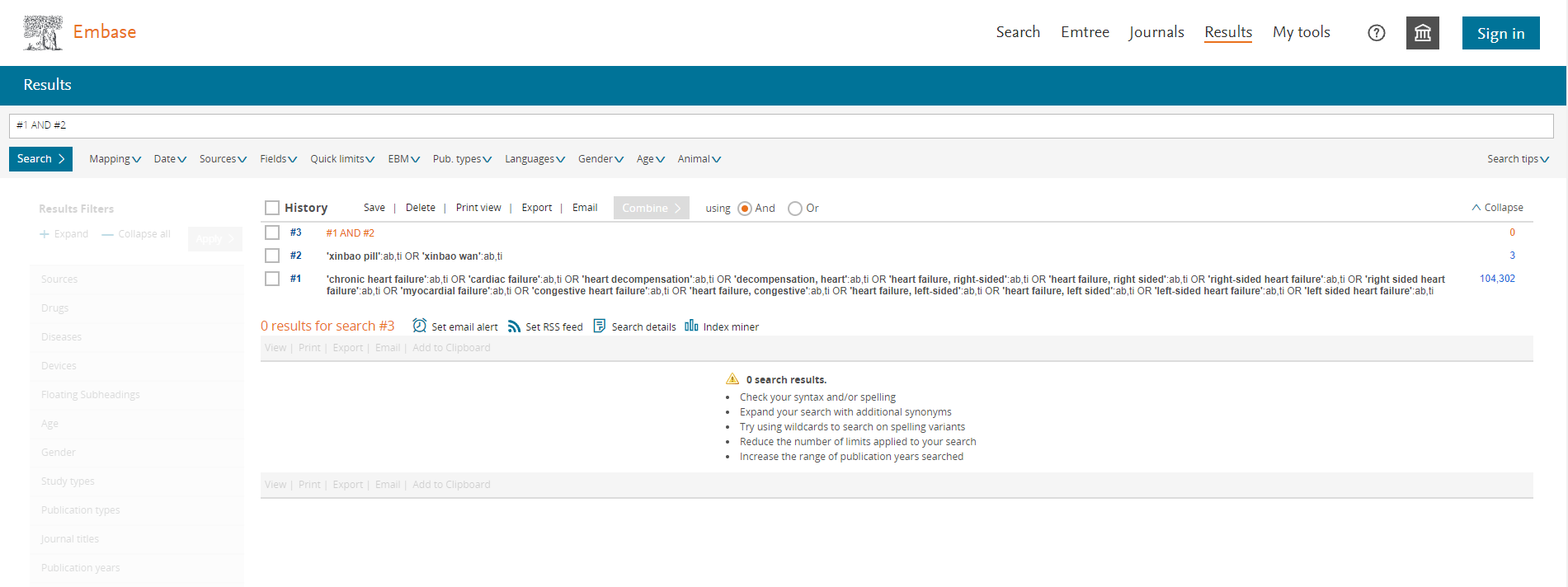


2.3 Cochrane Library (**<https://www.cochranelibrary.com/>**)

**Search date:December 19, 2021 Beijing time**

| ID | Search | Hits |
| --- | --- | --- |
| #1 | MeSH descriptor: [Heart Failure] explode all trees | 10145 |
| #2 | (Cardiac Failure OR Heart Decompensation OR Decompensation, Heart OR Heart Failure, Right-Sided OR Heart Failure, Right Sided OR Right-Sided Heart Failure OR Right Sided Heart Failure OR Myocardial Failure OR Congestive Heart Failure OR Heart Failure, Congestive OR Heart Failure, Left-Sided OR Heart Failure, Left Sided OR Left-Sided Heart Failure OR Left Sided Heart Failure):ti,ab,kw | 25275 |
| #3 | (Xinbao wan OR xinbao pill):ti,ab,kw | 4 |
| #4 | #1 OR #2 | 29682 |
| #5 | #3 and #4 | 2 |

**
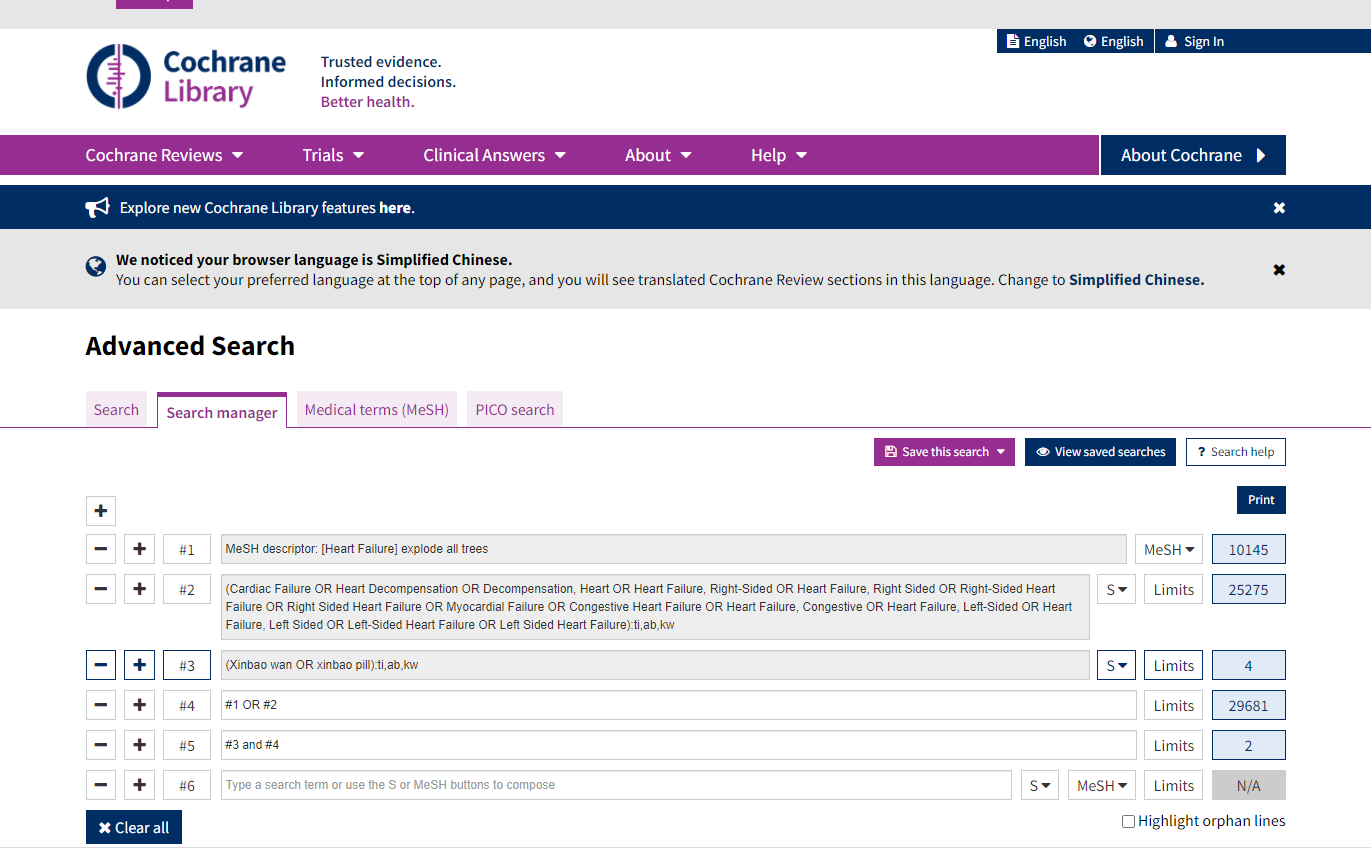
**

Clinical study of Xinbao pill in the treatment of chronic heart failure

ChiCTR‐IPR‐17011876

https://trialsearch.who.int/Trial2.aspx?TrialID=ChiCTR-IPR-17011876, 2017

Clinical study of Xinbao pill in the treatment of chronic heart failure (CHF) with deficiencysyndrome of heart and kidney yang

ChiCTR2000038492

https://trialsearch.who.int/Trial2.aspx?TrialID=ChiCTR2000038492, 2020

2.4 CNKI (https://www.cnki.net/)

**Search date:December 19, 2021 Beijing time**

**Search strategies: SU='心宝丸'*'心力衰竭'**


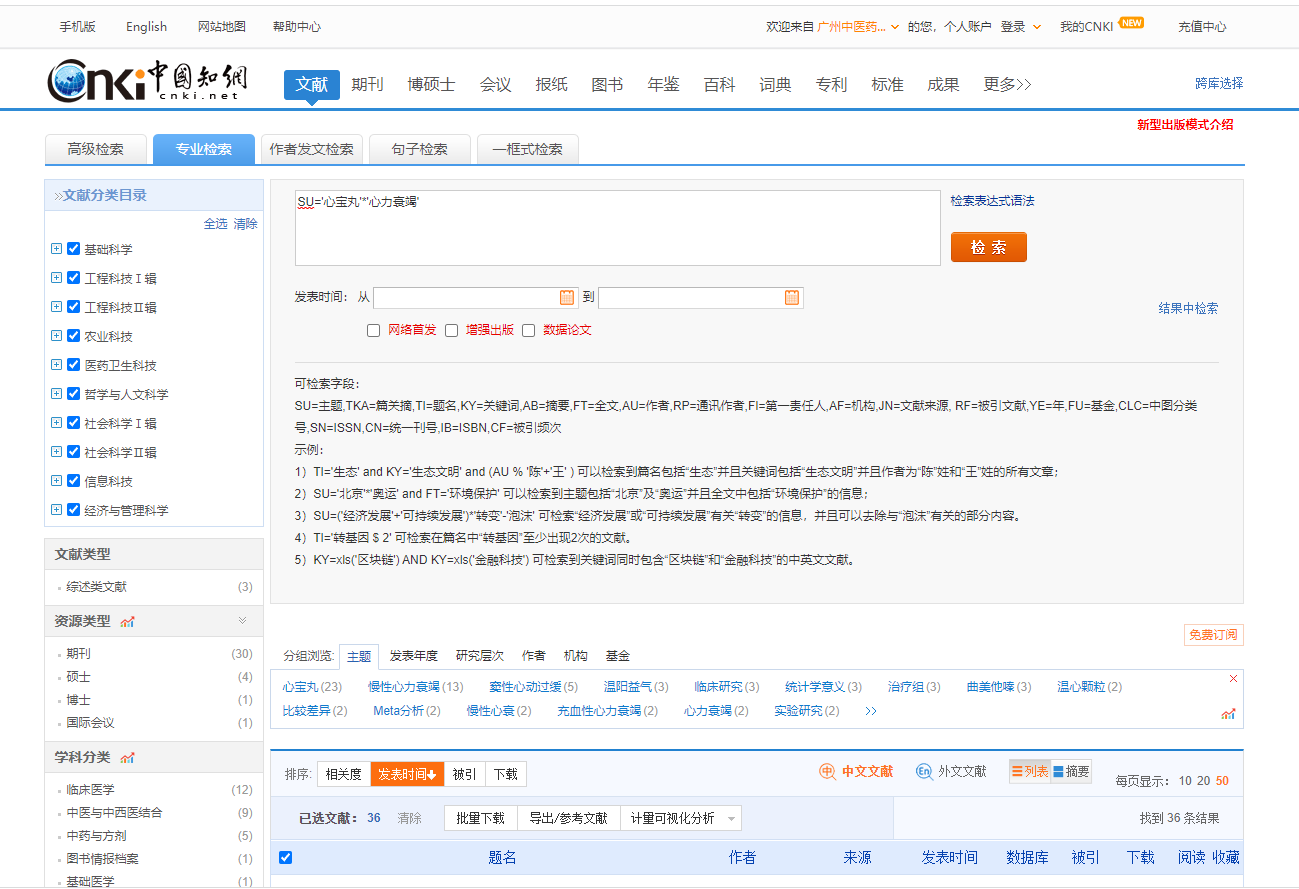


[1]何培坤,顾民华,徐煜凌,刘晓瑜,洪睦铿,贾钰华.基于PI3K/Akt/GSK3β信号通路研究心宝丸对慢性心力衰竭大鼠心肌肥厚的影响[J].中国中西医结合杂志,2020,40(12):1471-1477.

[2]吴继雷,冯秀芝,王凤荣.心宝丸治疗心肌梗死PCI术后合并慢性心力衰竭的疗效观察[J].中国实用医药,2020,15(16):104-106.

[3]卢方贵,徐义,黄素素.厄贝沙坦联合心宝丸治疗老年慢性心力衰竭合并窦性心动过缓的临床效果分析[J].北方药学,2020,17(06):116-117.

[4]胡芳,沈金峰,刘中勇.慢性心力衰竭的中医药研究进展[J].广州中医药大学学报,2020,37(06):1202-1206.

[5]李尊江. 基于网络药理学研究心宝丸对大鼠H9c2心肌细胞损伤的药效[D].广州中医药大学,2020.

[6]王渊,李天力,任骞,王宁勃,蒲凤兰,王显.心宝丸联合常规西药治疗慢性心力衰竭伴窦性心动过缓的Meta分析[J].中国循证心血管医学杂志,2020,12(03):264-268+280.

[7]徐静,齐惠英.心宝丸联合卡维地洛治疗慢性心力衰竭的临床研究[J].现代药物与临床,2019,34(12):3539-3542.

[8]李秋君.心宝丸联合曲美他嗪治疗慢性心衰合并窦性心动过缓的临床效果评价[J].中西医结合心血管病电子杂志,2019,7(27):4-5.

[9]王治国,郑艳妮,沙海旺.心宝丸联合米力农治疗终末期心力衰竭的临床研究[J].现代药物与临床,2019,34(09):2603-2608.

[10]李静,吕国芬.心宝丸联合美托洛尔治疗慢性心力衰竭的临床研究[J].现代药物与临床,2019,34(05):1294-1297.

[11]于潇潇. 中药治疗慢性心力衰竭临床试验方案设计[D].广州中医药大学,2019.

[12]赵雪莲,郭文娟,郑铎.厄贝沙坦联合心宝丸治疗老年慢性心力衰竭合幵窦性心动过缓的临床效果[J].中国药物经济学,2019,14(01):48-51.

[13]于潇潇,周仙仕,吴炎华,欧爱华,丁邦晗.心宝丸治疗慢性心力衰竭的系统评价[J].广州中医药大学学报,2019,36(02):153-159.

[14]李尊江,于潇潇,王冬梅,古江勇,刘云涛,丁邦晗,吴晓新.基于网络药理学研究心宝丸治疗慢性心力衰竭的作用机制[J].中药新药与临床药理,2018,29(06):768-774.

[15]闵红燕,江柳,张密浓.心宝丸对老年慢性心功能不全患者运动耐量和B型脑钠肽前体水平的影响[J].中国实用医药,2018,13(32):16-18.

[16]王彪,尤冬霞,赵林凤.厄贝沙坦联合心宝丸治疗老年慢性心力衰竭合并窦性心动过缓的效果[J].中西医结合心血管病电子杂志,2018,6(22):113.

[17]陈振岭,郑玉水,李为,芦伟.厄贝沙坦联合心宝丸治疗老年慢性心力衰竭合并窦性心动过缓的临床效果[J].中国医药导报,2018,15(02):54-57.

[18]张碧华,杨莉萍,唐鹏.慢性心力衰竭与中医相关病证的渊源与发展[J].中国中西医结合杂志,2018,38(05):633-635.

[19]高冶,王学磊,薛敏.心宝丸治疗合并心动过缓的慢性心力衰竭的疗效观察[J].内蒙古医学杂志,2016,48(04):471-472.

[20]张莹莹.心宝丸联合曲美他嗪治疗慢性心衰合并窦性心动过缓的临床疗效观察[J].湖北中医杂志,2016,38(04):3-5.

[21]黄亚莉.心宝丸联合比索洛尔治疗冠心病心力衰竭疗效观察[J].陕西中医,2014,35(11):1480-1481.

[22]魏勇,周志奇,邵静.心宝丸治疗慢性收缩性心力衰竭60例临床观察[J].中医临床研究,2014,6(13):63-64.

[23]陈勇鹏.卡维地洛联合曲美他嗪 心宝丸治疗慢性心力衰竭疗效观察[J].基层医学论坛,2013,17(01):4-6.

[24]陈勇鹏.比索洛尔联合心宝丸治疗冠心病心力衰竭效果观察[J].基层医学论坛,2012,16(19):2516-2517.

[25]刘春香,毛静远,王贤良.芪苈强心胶囊的临床应用及机制研究概况[J].时珍国医国药,2010,21(09):2349-2351.

[26]马民,张桂娟,莫宏波,马义,李德辉.活血温阳益气中药复方对大鼠充血性心力衰竭的影响及机制研究[J].中国病理生理杂志,2009,25(11):2126-2130.

[27]马民,张桂娟,莫宏波,马义,李德辉. 活血温阳益气中药复方对大鼠充血性心力衰竭的影响及机制研究[C]. 中国病理生理学会、国际病理生理学会.2009年国际病理生理学教学研讨会论文集.中国病理生理学会、国际病理生理学会:中国病理生理学会,2009:52-56.

[28]姜冬云. 温心颗粒治疗慢性充血性心力衰竭作用机理研究[D].成都中医药大学,2008.

[29]单亮,陈光亮.中医药治疗慢性充血性心力衰竭的研究进展[J].安徽中医学院学报,2007(04):63-64.

[30]仲伟琴,张晓莉,王保和.加参强心方治疗冠心病心力衰竭气虚血瘀证疗效观察[J].河南中医,2007(07):23-24.

[31]高晔. 茯苓四逆汤加味方治疗充血性心力衰竭的实验研究[D].成都中医药大学,2006.

[32]高霞. 温心颗粒益气温阳、活血化瘀作用实验研究[D].成都中医药大学,2006.

[33]郑偕扣,孙利,孙兰军,赵英强.强心力胶囊结合西药治疗慢性心力衰竭46例临床疗效观察[J].北京中医,2006(03):190-191.

[34]马民,莫宏波,陈利国,曹勇.活血益气复方治疗充血性心力衰竭的实验研究[J].四川大学学报(医学版),2005(05):736-737+753.

[35]胡有志,石杰,向楠,李大锋,肖遥,冯德勋.强心力胶囊治疗慢性充血性心力衰竭的临床研究[J].湖北中医杂志,2005(09):21-22.

[36]于凯成.心力衰竭的中医药治疗[J].中国社区医师,1996(12):21-25.

## **2.5 Wanfang data**

**Search date:December 19, 2021 Beijing time**

**Search strategies:主题:("心宝丸" and "心力衰竭")**


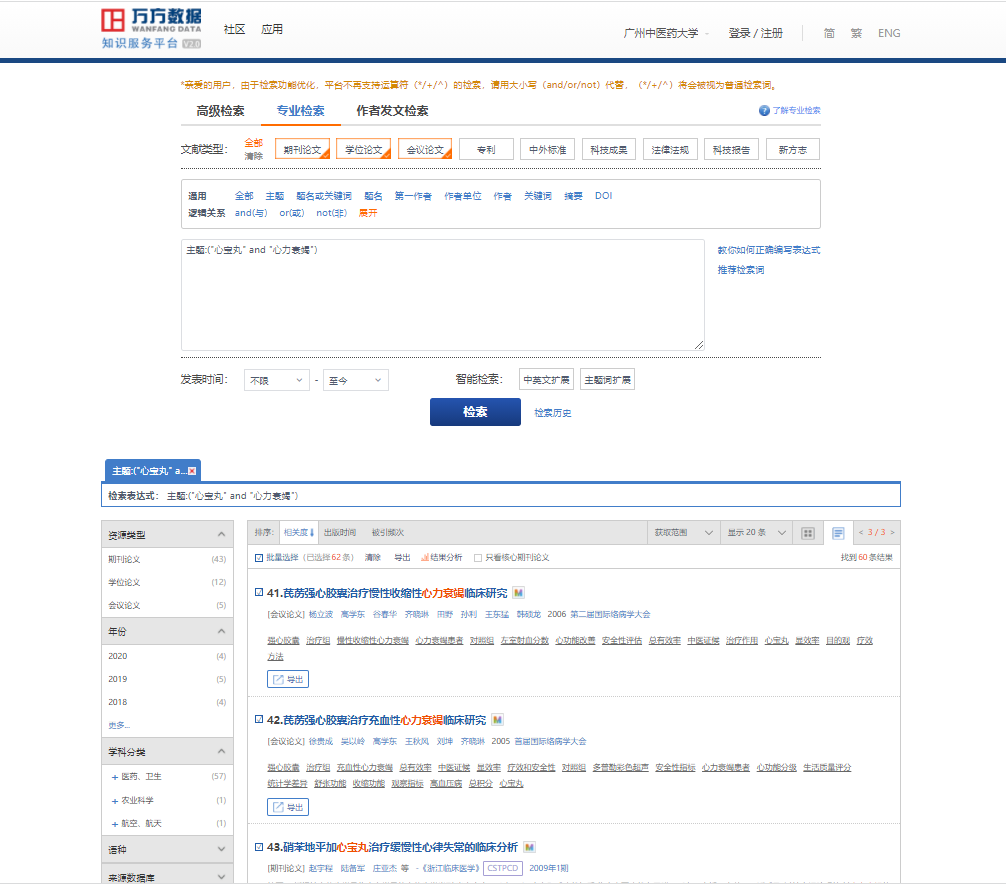


[1] 魏勇,周志奇,邵静. 心宝丸治疗慢性收缩性心力衰竭60例临床观察[J]. 中医临床研究,2014(13):63-64. DOI:10.3969/j.issn.1674-7860.2014.13.038.

[2] 谷仿丽. 心衰康颗粒对慢性心力衰竭大鼠的作用及其机制研究[D]. 安徽:安徽中医学院,2006.

[3] 刘强,李萌,钟志国,等. 强心利水方对慢性心衰大鼠神经内分泌的影响[J]. 中国中医基础医学杂志,2008,14(8):592-593. DOI:10.3969/j.issn.1006-3250.2008.08.013.

[4] 郭秋红,赵淑明,张一昕,等. 葶苈生脉方对慢性心力衰竭大鼠血流动力学的影响[J]. 河北中医药学报,2009,24(2):5-6. DOI:10.3969/j.issn.1007-5615.2009.02.002.

[5] 郭秋红,李昌,周桦,等. 葶苈生脉方对心衰大鼠血浆血管紧张素Ⅱ和醛固酮的影响[J]. 时珍国医国药,2010,21(9):2240-2241. DOI:10.3969/j.issn.1008-0805.2010.09.053.

[6] 张金兰,张辉,鲍新民,等. 人参强心滴丸对慢性心衰大鼠血浆ET-1和CGRP的影响[J]. 河北中医药学报,2011,26(1):36-37. DOI:10.3969/j.issn.1007-5615.2011.01.024.

[7] 赵淑明,张一昕,郭秋红,等. 葶苈生脉饮对慢性心衰大鼠心肌MMP-9、TIMP-1表达及胶原影响的研究[J]. 中药药理与临床,2009,25(2):91-92.

[8] 郑偕扣,孙利,孙兰军,等. 强心力胶囊结合西药治疗慢性心力衰竭46例临床疗效观察[J]. 北京中医,2006,25(3):190-191. DOI:10.3969/j.issn.1674-1307.2006.03.036.

[9] 牛平平. 暖心胶囊治疗慢性心衰的临床与实验机制研究[D]. 广东:广州中医药大学,2011.

[10] 李萌. 强心活血利水方对慢性心衰大鼠神经内分泌的影响[D]. 广东:广东医学院,2009. DOI:10.7666/d.y2013071.

[11] 王保和,孙兰军,刘玉梅,等. 加参强心方治疗充血性心力衰竭的临床研究[C]. //中国·天津第五届国际中医药学术研讨会暨第九届国际针灸学术交流会论文集. 2006:130-131.

[12] 郑偕扣. 强心力胶囊治疗慢性充血性心力衰竭（阳气虚乏，血瘀水停证）临床研究[D]. 天津中医药大学,2005.

[13] 郭秋红,彭雪梅,赵淑明,等. 葶苈生脉方对心衰大鼠AngⅡ及其受体信号传导的影响[J]. 中药新药与临床药理,2010,21(3):260-262.

[14] 翟卷平,郭秋红,王卓,等. 葶苈生脉方对心衰大鼠心肌细胞凋亡的影响[J]. 河北中医药学报,2010,25(3):5-6. DOI:10.3969/j.issn.1007-5615.2010.03.002.

[15] 郭秋红,刘敬颇,赵淑明,等. 葶苈生脉方对心衰大鼠RAAS及心肌组织AT1 mRNA表达的影响[J]. 中华中医药杂志,2010,25(10):1654-1656.

[16] 郭秋红,张一昕,赵淑明,等. 葶苈生脉方对心衰大鼠心肌纤维化及TGF-β_1表达的影响[J]. 中华中医药杂志,2010,25(4):585-587.

[17] 邹旭,牛平平,姚耿圳,等. 暖心胶囊对心衰大鼠肌浆网Ca2+-ATP酶、血浆AngⅡ、ALD以及血流动力学的影响[J]. 新中医,2011,43(11):110-112.

[18] 孙阳,朱明军,李彬,等. 芪参益气滴丸治疗心衰现状探讨[J]. 中国中医药现代远程教育,2019,17(3):54-56. DOI:10.3969/j.issn.1672-2779.2019.03.022.

[19] 杨林. 参附益心颗粒治疗冠心病心力衰竭(气虚阳虚兼血瘀水阻证)的临床观察[D]. 辽宁:辽宁中医药大学,2009. DOI:10.7666/d.y1587089.

[20] 周桦. 葶苈生脉方对心衰大鼠AngⅡ及其受体信号传导的影响[D]. 河北:河北医科大学,2009. DOI:10.7666/d.y1637601.

[21] 赵明明,王海蓉,熊峰,等. 稳心颗粒联合美托洛尔治疗心房颤动有效性和安全性的Meta分析[J]. 中国医药,2014,9(4):458-463. DOI:10.3760/cma.j.issn.1673-4777.2014.04.003.

[22] 王渊,李天力,任骞,等. 心宝丸联合常规西药治疗慢性心力衰竭伴窦性心动过缓的Meta分析[J]. 中国循证心血管医学杂志,2020,12(3):264-268,280. DOI:10.3969/j.issn.1674-4055.2020.03.03.

[23] 卢方贵,徐义,黄素素. 厄贝沙坦联合心宝丸治疗老年慢性心力衰竭合并窦性心动过缓的临床效果分析[J]. 北方药学,2020,17(6):116-117.

[24] 吴继雷,冯秀芝,王凤荣. 心宝丸治疗心肌梗死PCI术后合并慢性心力衰竭的疗效观察[J]. 中国实用医药,2020,15(16):104-106. DOI:10.14163/j.cnki.11-5547/r.2020.16.044.

[25] 何培坤,顾民华,徐煜凌,等. 基于PI3K/Akt/GSK3β信号通路研究心宝丸对慢性心力衰竭大鼠心肌肥厚的影响[J]. 中国中西医结合杂志,2020,40(12):1471-1477. DOI:10.7661/j.cjim.20200814.034.

[26] 赵雪莲,郭文娟,郑铎. 厄贝沙坦联合心宝丸治疗老年慢性心力衰竭合幵窦性心动过缓的临床效果[J]. 中国药物经济学,2019,14(1):48-51. DOI:10.12010/j.issn.1673-5846.2019.01.011.

[27] 陈振岭,郑玉水,李为,等. 厄贝沙坦联合心宝丸治疗老年慢性心力衰竭合并窦性心动过缓的临床效果[J]. 中国医药导报,2018,15(2):54-57.

[28] 王彪,尤冬霞,赵林凤. 厄贝沙坦联合心宝丸治疗老年慢性心力衰竭合并窦性心动过缓的效果[J]. 中西医结合心血管病电子杂志,2018,6(22):113. DOI:10.3969/j.issn.2095-6681.2018.22.082.

[29] 刘林强,王玲,孙雷雷. 心宝丸治疗终末期心力衰竭的疗效观察[J]. 中国实用乡村医生杂志,2015(5):55-56. DOI:10.3969/j.issn.1672-7185.2015.05.033.

[30] 黄亚莉. 心宝丸联合比索洛尔治疗冠心病心力衰竭疗效观察[J]. 陕西中医,2014(11):1480-1481. DOI:10.3969/j.issn.1000-7369.2014.11.023.

[31] 高冶,王学磊,薛敏. 心宝丸治疗合并心动过缓的慢性心力衰竭的疗效观察[J]. 内蒙古医学杂志,2016,48(4):471-472. DOI:10.16096/J.cnki.nmgyxzz.2016.48.04.034.

[32] 陈勇鹏. 比索洛尔联合心宝丸治疗冠心病心力衰竭效果观察[J]. 基层医学论坛,2012,16(19):2516-2517. DOI:10.3969/j.issn.1672-1721.2012.19.049.

[33] 李尊江,于潇潇,王冬梅,等. 基于网络药理学研究心宝丸治疗慢性心力衰竭的作用机制[J]. 中药新药与临床药理,2018,29(6):768-774. DOI:10.19378/j.issn.1003-9783.2018.06.016.

[34] 李静,吕国芬. 心宝丸联合美托洛尔治疗慢性心力衰竭的临床研究[J]. 现代药物与临床,2019,34(5):1294-1297. DOI:10.7501/j.issn.1674-5515.2019.05.006.

[35] 王治国,郑艳妮,沙海旺. 心宝丸联合米力农治疗终末期心力衰竭的临床研究[J]. 现代药物与临床,2019,34(9):2603-2608. DOI:10.7501/j.issn.1674-5515.2019.09.006.

[36] 郭秋红,张一昕,赵淑明,等. 葶苈生脉方对充血性心力衰竭大鼠血浆内皮素-1、降钙素基因相关肽的影响[J]. 中国老年学杂志,2009,29(11):1338-1340. DOI:10.3969/j.issn.1005-9202.2009.11.007.

[37] 徐静,齐惠英. 心宝丸联合卡维地洛治疗慢性心力衰竭的临床研究[J]. 现代药物与临床,2019,34(12):3539-3542. DOI:10.7501/j.issn.1674-5515.2019.12.008.

[38] 张志霞,张一昕,李国川,等. 人参强心滴丸对充血性心力衰竭大鼠血浆心钠素的影响[J]. 中国中医基础医学杂志,2007,13(7):517-518. DOI:10.3969/j.issn.1006-3250.2007.07.014.

[39] 张志霞,张一昕,李国川,等. 人参强心滴丸对充血性心力衰竭大鼠血清TNF-α和IL-1含量的影响[J]. 中华中医药杂志,2007,22(8):549-551. DOI:10.3969/j.issn.1673-1727.2007.08.016.

[40] 高新明. 加参强心方治疗冠心病心力衰竭气虚血瘀证疗效观察[J]. 临床医药文献电子杂志,2018,5(77):30. DOI:10.3877/j.issn.2095-8242.2018.77.025.

[41] 史海锋. 芪苈强心胶囊治疗慢性收缩性心力衰竭的临床疗效[J]. 医疗装备,2016,29(16):161-161,162. DOI:10.3969/j.issn.1002-2376.2016.16.125.

[42] 仲伟琴,张晓莉,王保和. 加参强心方治疗冠心病心力衰竭气虚血瘀证疗效观察[J]. 河南中医,2007,27(7):23-24. DOI:10.3969/j.issn.1003-5028.2007.07.011.

[43] 乔崇. 芪苈强心胶囊治疗慢性收缩性心力衰竭的疗效探讨[J]. 中西医结合心血管病电子杂志,2016,4(13):85-85,88.

[44] 刘莉,田淇元,金娟. 利心I号对充血性心力衰竭大鼠心肌细胞Bax、Bcl-2表达的影响[J]. 中医药学报,2008,36(3):22-23. DOI:10.3969/j.issn.1002-2392.2008.03.008.

[45] 徐贵成,王秋风,刘坤,等. 芪苈强心胶囊治疗慢性收缩性心力衰竭的临床研究[J]. 疑难病杂志,2008,7(5):262-265. DOI:10.3969/j.issn.1671-6450.2008.05.003.

[46] 杨立波,陈延军,张喜芬. 芪苈强心胶囊治疗慢性收缩性心力衰竭的临床研究[J]. 疑难病杂志,2010,9(11):805-807. DOI:10.3969/j.issn.1671-6450.2010.11.001.

[47] 吴以岭,谷春华,徐贵成,等. 芪苈强心胶囊治疗慢性心力衰竭随机双盲、多中心临床研究[J]. 疑难病杂志,2007,6(5):263-266. DOI:10.3969/j.issn.1671-6450.2007.05.003.

[48] 雷峥. 芪苈强心胶囊治疗慢性心力衰竭[J]. 中国医药指南,2008,6(15):118-119. DOI:10.3969/j.issn.1671-8194.2008.15.072.

[49] 胡有志,石杰,向楠,等. 强心力胶囊治疗慢性充血性心力衰竭的临床研究[J]. 湖北中医杂志,2005,27(9):21-22. DOI:10.3969/j.issn.1000-0704.2005.09.010.

[50] 于潇潇. 中药治疗慢性心力衰竭临床试验方案设计--以心宝丸临床试验为例[D]. 广东:广州中医药大学,2019.

[51] 吴以岭,谷春华,徐贵成,等. 芪苈强心胶囊治疗慢性心力衰竭随机双盲、多中心临床研究[C]. //第十届国际络病学大会论文集. 2014:578-581.

[52] 吴以岭,谷春华,徐贵成,等. 芪苈强心胶囊治疗慢性心力衰竭随机双盲、多中心临床研究[C]. //中华中医药学会第三届国际络病学大会论文集. 2007:459-462.

[53] 李丹. 利心Ⅰ号对充血性心力衰竭大鼠血浆内皮素及心钠素的影响[D]. 黑龙江:黑龙江中医药大学,2007. DOI:10.7666/d.y1129475.

[54] 孟繁蕴. 强心通脉饮治疗充血性心力衰竭的临床及实验研究[D]. 山东:山东中医药大学,2003. DOI:10.7666/d.Y496417.

[55] 杨立波,高学东,谷春华,等. 芪苈强心胶囊治疗慢性收缩性心力衰竭临床研究[C]. //络病学基础与临床研究(2)--第二届国际络病学大会论文集. 2006.

[56] 徐贵成,吴以岭,高学东,等. 芪苈强心胶囊治疗充血性心力衰竭临床研究[C]. //首届国际络病学大会论文集. 2005.

[57] 赵宇程,陆备军,庄亚杰,等. 硝苯地平加心宝丸治疗缓慢性心律失常的临床分析[J]. 浙江临床医学,2009,11(1):73-74. DOI:10.3969/j.issn.1008-7664.2009.01.039.

[58] 赵淑明,郭秋红,张志良,等. 葶苈生脉饮对压力负荷大鼠心肌组织MMP-9及TIMP-1表达的影响[J]. 中国老年学杂志,2009,29(16):2067-2069. DOI:10.3969/j.issn.1005-9202.2009.16.029.

[59] 刘莉,邹国良,索传涛. 利心Ⅰ号对CHF大鼠血流动力学、心肌细胞ICAM-1基因蛋白表达的影响[J]. 心脏杂志,2007,19(2):166-169. DOI:10.3969/j.issn.1009-7236.2007.02.012.

[60] 黑春潮. 葶苈生脉方对心衰大鼠心肌细胞凋亡及相关调控基因的影响[D]. 河北:河北医科大学,2009. DOI:10.7666/d.y1636967.

[61] 苗冬雪. 人参强心滴丸对阿霉素心衰大鼠心肌细胞凋亡的影响[D]. 河北:河北医科大学,2007. DOI:10.7666/d.y1156640.

[62] 张志良. 慢性心衰大鼠心肌MMP-9、TIMP-1的表达及葶苈生脉方对其影响的研究[D]. 河北:河北医科大学,2009. DOI:10.7666/d.y1636480.

2.6 Cqvip（http://www.cqvip.com/）

**Search date:December 19, 2021 Beijing time**

**Search strategies: M=心宝丸 AND 心力衰竭**


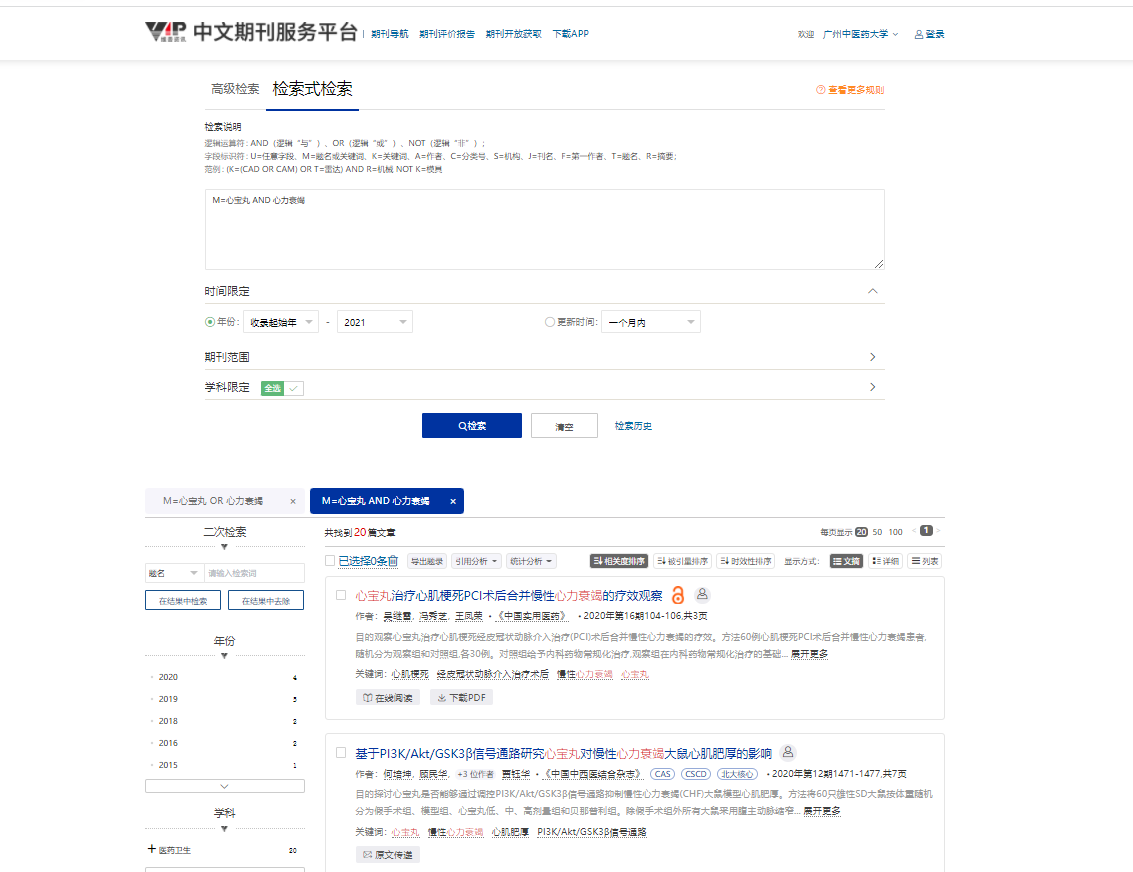


[1]吴继雷,冯秀芝,王凤荣.心宝丸治疗心肌梗死PCI术后合并慢性心力衰竭的疗效观察[J].中国实用医药,2020,15(16):104-106.

[2]何培坤,顾民华,徐煜凌,刘晓瑜,洪睦铿,贾钰华.基于PI3K/Akt/GSK3β信号通路研究心宝丸对慢性心力衰竭大鼠心肌肥厚的影响[J].中国中西医结合杂志,2020,40(12):1471-1477.

[3]王渊,李天力,任骞,王宁勃,蒲凤兰,王显.心宝丸联合常规西药治疗慢性心力衰竭伴窦性心动过缓的Meta分析[J].中国循证心血管医学杂志,2020,12(3):264-268+280.

[4]卢方贵,徐义,黄素素.厄贝沙坦联合心宝丸治疗老年慢性心力衰竭合并窦性心动过缓的临床效果分析[J].北方药学,2020,17(6):116-117.

[5]赵雪莲,郭文娟,郑铎.厄贝沙坦联合心宝丸治疗老年慢性心力衰竭合幵窦性心动过缓的临床效果[J].中国药物经济学,2019(1):48-51.

[6]徐静,齐惠英.心宝丸联合卡维地洛治疗慢性心力衰竭的临床研究[J].现代药物与临床,2019,34(12):3539-3542.

[7]于潇潇,周仙仕,吴炎华,欧爱华,丁邦晗.心宝丸治疗慢性心力衰竭的系统评价[J].广州中医药大学学报,2019,36(2):153-159.

[8]王治国,郑艳妮,沙海旺.心宝丸联合米力农治疗终末期心力衰竭的临床研究[J].现代药物与临床,2019(9):2603-2608.

[9]李静,吕国芬.心宝丸联合美托洛尔治疗慢性心力衰竭的临床研究[J].现代药物与临床,2019,34(5):1294-1297.

[10]李尊江,于潇潇,王冬梅,古江勇,刘云涛,丁邦晗,吴晓新.基于网络药理学研究心宝丸治疗慢性心力衰竭的作用机制[J].中药新药与临床药理,2018,29(6):768-774.

[11]陈振岭,郑玉水,李为,芦伟.厄贝沙坦联合心宝丸治疗老年慢性心力衰竭合并窦性心动过缓的临床效果[J].中国医药导报,2018,15(2):54-57.

[12]高冶,王学磊,薛敏.心宝丸治疗合并心动过缓的慢性心力衰竭的疗效观察[J].内蒙古医学杂志,2016(4):471-472.

[13]刘林强,王玲,孙雷雷.心宝丸治疗终末期心力衰竭的疗效观察[J].中国实用乡村医生杂志,2015(5):55-56.

[14]黄亚莉.心宝丸联合比索洛尔治疗冠心病心力衰竭疗效观察[J].陕西中医,2014(11):1480-1481.

[15]魏勇,周志奇,邵静.心宝丸治疗慢性收缩性心力衰竭60例临床观察[J].中医临床研究,2014,6(13):63-64.

[16]陈勇鹏.卡维地洛联合曲美他嗪 心宝丸治疗慢性心力衰竭疗效观察[J].基层医学论坛,2013(1):4-6.

[17]陈勇鹏.比索洛尔联合心宝丸治疗冠心病心力衰竭效果观察[J].基层医学论坛,2012,16(19):2516-2517.

[18]赵宇程,陆备军,庄亚杰,施伟国.硝苯地平加心宝丸治疗缓慢性心律失常的临床分析[J].浙江临床医学,2009,11(1):73-74.

[19]仲伟琴,张晓莉,王保和.加参强心方治疗冠心病心力衰竭气虚血瘀证疗效观察[J].河南中医,2007,27(7):23-24.

[20]张莹莹.心宝丸联合曲美他嗪治疗慢性心衰合并窦性心动过缓的临床疗效观察[J].湖北中医杂志,2016,38(4):3-5.

2.7 ClinicalTrials.gov (**<https://clinicaltrials.gov/>**)

**Search date:December 19, 2021 Beijing time**

**
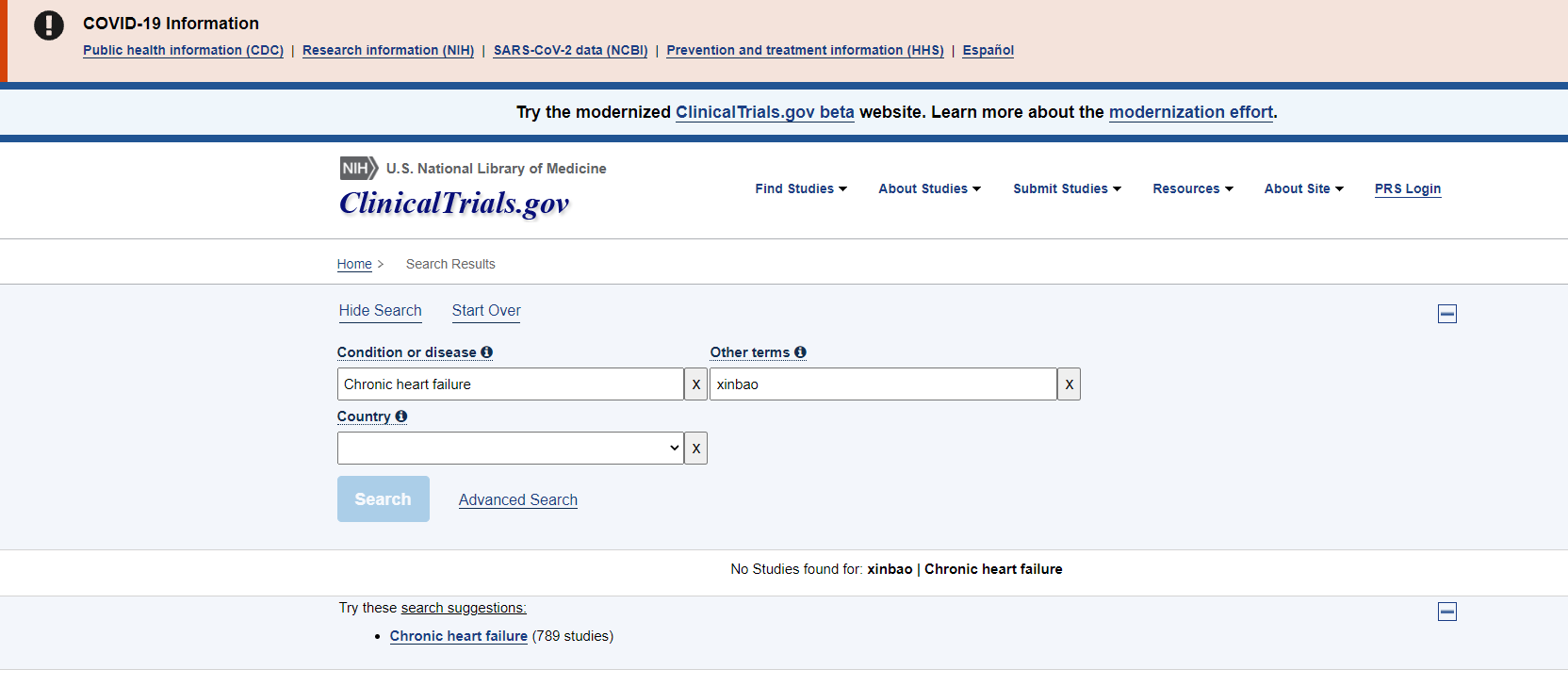
**

2.8 CHiCTR (http://www.chictr.org.cn/index.aspx)

**Search date:December 19, 2021 Beijing time**

**
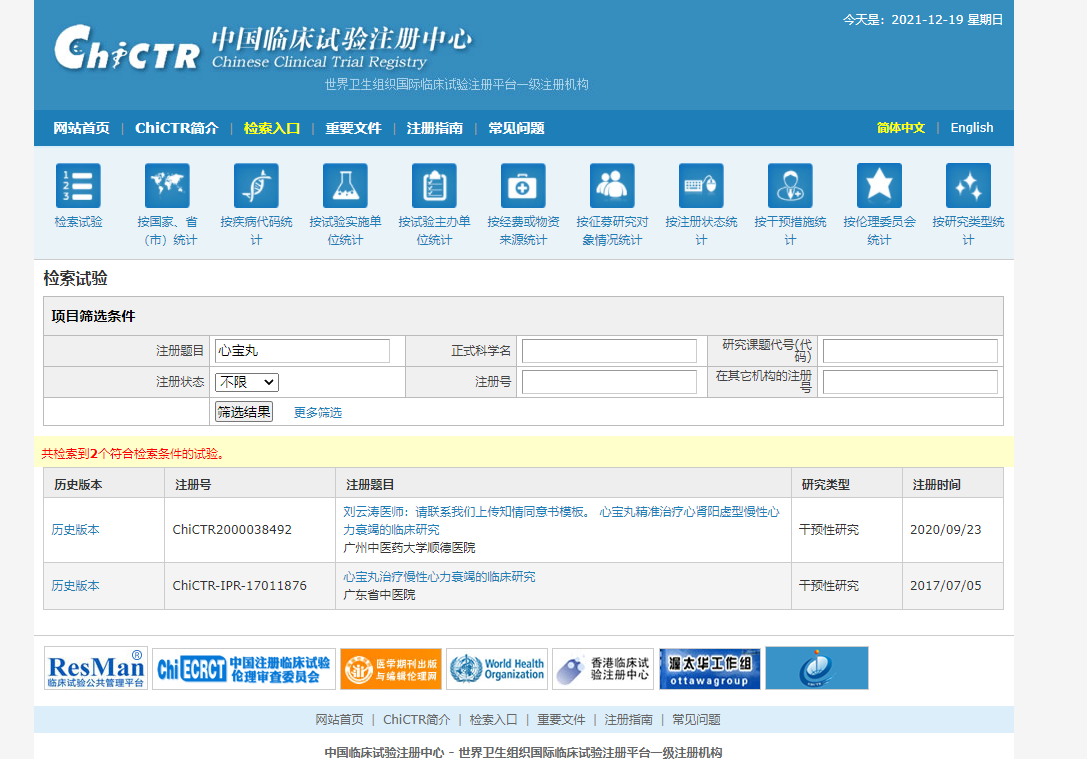
**

# Supplementary File S3. A list of excluded studies by reading title and abstract.

**Study design:**

1. 何培坤,顾民华,徐煜凌,等. 基于PI3K/Akt/GSK3β信号通路研究心宝丸对慢性心力衰竭大鼠心肌肥厚的影响[J]. 中国中西医结合杂志,2020,40(12):1471-1477. DOI:10.7661/j.cjim.20200814.034.
2. 胡芳,沈金峰,刘中勇.慢性心力衰竭的中医药研究进展[J].广州中医药大学学报,2020,37(06):1202-1206.
3. 李尊江,于潇潇,王冬梅,等. 基于网络药理学研究心宝丸治疗慢性心力衰竭的作用机制[J]. 中药新药与临床药理,2018,29(6):768-774. DOI:10.19378/j.issn.1003-9783.2018.06.016.
4. 李尊江,于潇潇,王冬梅,古江勇,刘云涛,丁邦晗,吴晓新.基于网络药理学研究心宝丸治疗慢性心力衰竭的作用机制[J].中药新药与临床药理,2018,29(6):768-774.
5. 李尊江. 基于网络药理学研究心宝丸对大鼠H9c2心肌细胞损伤的药效[D].广州中医药大学,2020.
6. 王渊,李天力,任骞,等. 心宝丸联合常规西药治疗慢性心力衰竭伴窦性心动过缓的Meta分析[J]. 中国循证心血管医学杂志,2020,12(3):264-268,280. DOI:10.3969/j.issn.1674-4055.2020.03.03.
7. 王渊,李天力,任骞,王宁勃,蒲凤兰,王显.心宝丸联合常规西药治疗慢性心力衰竭伴窦性心动过缓的Meta分析[J].中国循证心血管医学杂志,2020,12(03):264-268+280.
8. 于潇潇,周仙仕,吴炎华,欧爱华,丁邦晗.心宝丸治疗慢性心力衰竭的系统评价[J].广州中医药大学学报,2019,36(2):153-159.
9. 张碧华,杨莉萍,唐鹏.慢性心力衰竭与中医相关病证的渊源与发展[J].中国中西医结合杂志,2018,38(05):633-635.
10. Clinical study of Xinbao pill in the treatment of chronic heart failure.ChiCTR‐IPR‐17011876 https://trialsearch.who.int/Trial2.aspx?TrialID=ChiCTR-IPR-17011876, 2017
11. Clinical study of Xinbao pill in the treatment of chronic heart failure (CHF) with deficiencysyndrome of heart and kidney yang.ChiCTR2000038492 https://trialsearch.who.int/Trial2.aspx?TrialID=ChiCTR2000038492, 2020

**Intervention:**

1. 单亮,陈光亮.中医药治疗慢性充血性心力衰竭的研究进展[J].安徽中医学院学报,2007(04):63-64.
2. 翟卷平,郭秋红,王卓,等. 葶苈生脉方对心衰大鼠心肌细胞凋亡的影响[J]. 河北中医药学报,2010,25(3):5-6. DOI:10.3969/j.issn.1007-5615.2010.03.002.
3. 高霞. 温心颗粒益气温阳、活血化瘀作用实验研究[D].成都中医药大学,2006.
4. 高新明. 加参强心方治疗冠心病心力衰竭气虚血瘀证疗效观察[J]. 临床医药文献电子杂志,2018,5(77):30. DOI:10.3877/j.issn.2095-8242.2018.77.025.
5. 高晔. 茯苓四逆汤加味方治疗充血性心力衰竭的实验研究[D].成都中医药大学,2006.
6. 谷仿丽. 心衰康颗粒对慢性心力衰竭大鼠的作用及其机制研究[D]. 安徽:安徽中医学院,2006.
7. 郭秋红,李昌,周桦,等. 葶苈生脉方对心衰大鼠血浆血管紧张素Ⅱ和醛固酮的影响[J]. 时珍国医国药,2010,21(9):2240-2241. DOI:10.3969/j.issn.1008-0805.2010.09.053.
8. 郭秋红,刘敬颇,赵淑明,等. 葶苈生脉方对心衰大鼠RAAS及心肌组织AT1 mRNA表达的影响[J]. 中华中医药杂志,2010,25(10):1654-1656.
9. 郭秋红,彭雪梅,赵淑明,等. 葶苈生脉方对心衰大鼠AngⅡ及其受体信号传导的影响[J]. 中药新药与临床药理,2010,21(3):260-262.
10. 郭秋红,张一昕,赵淑明,等. 葶苈生脉方对充血性心力衰竭大鼠血浆内皮素-1、降钙素基因相关肽的影响[J]. 中国老年学杂志,2009,29(11):1338-1340. DOI:10.3969/j.issn.1005-9202.2009.11.007.
11. 郭秋红,张一昕,赵淑明,等. 葶苈生脉方对心衰大鼠心肌纤维化及TGF-β_1表达的影响[J]. 中华中医药杂志,2010,25(4):585-587.
12. 郭秋红,赵淑明,张一昕,等. 葶苈生脉方对慢性心力衰竭大鼠血流动力学的影响[J]. 河北中医药学报,2009,24(2):5-6. DOI:10.3969/j.issn.1007-5615.2009.02.002.
13. 黑春潮. 葶苈生脉方对心衰大鼠心肌细胞凋亡及相关调控基因的影响[D]. 河北:河北医科大学,2009. DOI:10.7666/d.y1636967.
14. 胡有志,石杰,向楠,等. 强心力胶囊治疗慢性充血性心力衰竭的临床研究[J]. 湖北中医杂志,2005,27(9):21-22. DOI:10.3969/j.issn.1000-0704.2005.09.010.
15. 雷峥. 芪苈强心胶囊治疗慢性心力衰竭[J]. 中国医药指南,2008,6(15):118-119. DOI:10.3969/j.issn.1671-8194.2008.15.072.
16. 李丹. 利心Ⅰ号对充血性心力衰竭大鼠血浆内皮素及心钠素的影响[D]. 黑龙江:黑龙江中医药大学,2007. DOI:10.7666/d.y1129475.
17. 李萌. 强心活血利水方对慢性心衰大鼠神经内分泌的影响[D]. 广东:广东医学院,2009. DOI:10.7666/d.y2013071.
18. 刘春香,毛静远,王贤良.芪苈强心胶囊的临床应用及机制研究概况[J].时珍国医国药,2010,21(09):2349-2351.
19. 刘莉,田淇元,金娟. 利心I号对充血性心力衰竭大鼠心肌细胞Bax、Bcl-2表达的影响[J]. 中医药学报,2008,36(3):22-23. DOI:10.3969/j.issn.1002-2392.2008.03.008.
20. 刘莉,邹国良,索传涛. 利心Ⅰ号对CHF大鼠血流动力学、心肌细胞ICAM-1基因蛋白表达的影响[J]. 心脏杂志,2007,19(2):166-169. DOI:10.3969/j.issn.1009-7236.2007.02.012.
21. 刘强,李萌,钟志国,等. 强心利水方对慢性心衰大鼠神经内分泌的影响[J]. 中国中医基础医学杂志,2008,14(8):592-593. DOI:10.3969/j.issn.1006-3250.2008.08.013.
22. 马民,莫宏波,陈利国,曹勇.活血益气复方治疗充血性心力衰竭的实验研究[J].四川大学学报(医学版),2005(05):736-737+753.
23. 马民,张桂娟,莫宏波,马义,李德辉. 活血温阳益气中药复方对大鼠充血性心力衰竭的影响及机制研究[C]. 中国病理生理学会、国际病理生理学会.2009年国际病理生理学教学研讨会论文集.中国病理生理学会、国际病理生理学会:中国病理生理学会,2009:52-56.
24. 马民,张桂娟,莫宏波,马义,李德辉.活血温阳益气中药复方对大鼠充血性心力衰竭的影响及机制研究[J].中国病理生理杂志,2009,25(11):2126-2130.
25. 孟繁蕴. 强心通脉饮治疗充血性心力衰竭的临床及实验研究[D]. 山东:山东中医药大学,2003. DOI:10.7666/d.Y496417.
26. 苗冬雪. 人参强心滴丸对阿霉素心衰大鼠心肌细胞凋亡的影响[D]. 河北:河北医科大学,2007. DOI:10.7666/d.y1156640.
27. 牛平平. 暖心胶囊治疗慢性心衰的临床与实验机制研究[D]. 广东:广州中医药大学,2011.
28. 乔崇. 芪苈强心胶囊治疗慢性收缩性心力衰竭的疗效探讨[J]. 中西医结合心血管病电子杂志,2016,4(13):85-85,88.
29. 史海锋. 芪苈强心胶囊治疗慢性收缩性心力衰竭的临床疗效[J]. 医疗装备,2016,29(16):161-161,162. DOI:10.3969/j.issn.1002-2376.2016.16.125.
30. 孙阳,朱明军,李彬,等. 芪参益气滴丸治疗心衰现状探讨[J]. 中国中医药现代远程教育,2019,17(3):54-56. DOI:10.3969/j.issn.1672-2779.2019.03.022.
31. 王保和,孙兰军,刘玉梅,等. 加参强心方治疗充血性心力衰竭的临床研究[C]. //中国·天津第五届国际中医药学术研讨会暨第九届国际针灸学术交流会论文集. 2006:130-131.
32. 吴以岭,谷春华,徐贵成,等. 芪苈强心胶囊治疗慢性心力衰竭随机双盲、多中心临床研究[C]. //第十届国际络病学大会论文集. 2014:578-581.
33. 吴以岭,谷春华,徐贵成,等. 芪苈强心胶囊治疗慢性心力衰竭随机双盲、多中心临床研究[C]. //中华中医药学会第三届国际络病学大会论文集. 2007:459-462.
34. 吴以岭,谷春华,徐贵成,等. 芪苈强心胶囊治疗慢性心力衰竭随机双盲、多中心临床研究[J]. 疑难病杂志,2007,6(5):263-266. DOI:10.3969/j.issn.1671-6450.2007.05.003.
35. 徐贵成,王秋风,刘坤,等. 芪苈强心胶囊治疗慢性收缩性心力衰竭的临床研究[J]. 疑难病杂志,2008,7(5):262-265. DOI:10.3969/j.issn.1671-6450.2008.05.003.
36. 徐贵成,吴以岭,高学东,等. 芪苈强心胶囊治疗充血性心力衰竭临床研究[C]. //首届国际络病学大会论文集. 2005.
37. 杨立波,陈延军,张喜芬. 芪苈强心胶囊治疗慢性收缩性心力衰竭的临床研究[J]. 疑难病杂志,2010,9(11):805-807. DOI:10.3969/j.issn.1671-6450.2010.11.001.
38. 杨立波,高学东,谷春华,等. 芪苈强心胶囊治疗慢性收缩性心力衰竭临床研究[C]. //络病学基础与临床研究(2)--第二届国际络病学大会论文集. 2006.
39. 杨林. 参附益心颗粒治疗冠心病心力衰竭(气虚阳虚兼血瘀水阻证)的临床观察[D]. 辽宁:辽宁中医药大学,2009. DOI:10.7666/d.y1587089.
40. 于潇潇. 中药治疗慢性心力衰竭临床试验方案设计[D].广州中医药大学,2019.
41. 张金兰,张辉,鲍新民,等. 人参强心滴丸对慢性心衰大鼠血浆ET-1和CGRP的影响[J]. 河北中医药学报,2011,26(1):36-37. DOI:10.3969/j.issn.1007-5615.2011.01.024.
42. 张志良. 慢性心衰大鼠心肌MMP-9、TIMP-1的表达及葶苈生脉方对其影响的研究[D]. 河北:河北医科大学,2009. DOI:10.7666/d.y1636480.
43. 张志霞,张一昕,李国川,等. 人参强心滴丸对充血性心力衰竭大鼠血浆心钠素的影响[J]. 中国中医基础医学杂志,2007,13(7):517-518. DOI:10.3969/j.issn.1006-3250.2007.07.014.
44. 张志霞,张一昕,李国川,等. 人参强心滴丸对充血性心力衰竭大鼠血清TNF-α和IL-1含量的影响[J]. 中华中医药杂志,2007,22(8):549-551. DOI:10.3969/j.issn.1673-1727.2007.08.016.
45. 赵明明,王海蓉,熊峰,等. 稳心颗粒联合美托洛尔治疗心房颤动有效性和安全性的Meta分析[J]. 中国医药,2014,9(4):458-463. DOI:10.3760/cma.j.issn.1673-4777.2014.04.003.
46. 赵淑明,郭秋红,张志良,等. 葶苈生脉饮对压力负荷大鼠心肌组织MMP-9及TIMP-1表达的影响[J]. 中国老年学杂志,2009,29(16):2067-2069. DOI:10.3969/j.issn.1005-9202.2009.16.029.
47. 赵淑明,张一昕,郭秋红,等. 葶苈生脉饮对慢性心衰大鼠心肌MMP-9、TIMP-1表达及胶原影响的研究[J]. 中药药理与临床,2009,25(2):91-92.
48. 郑偕扣,孙利,孙兰军,等. 强心力胶囊结合西药治疗慢性心力衰竭46例临床疗效观察[J]. 北京中医,2006,25(3):190-191. DOI:10.3969/j.issn.1674-1307.2006.03.036.
49. 郑偕扣. 强心力胶囊治疗慢性充血性心力衰竭（阳气虚乏，血瘀水停证）临床研究[D]. 天津中医药大学,2005.
50. 仲伟琴,张晓莉,王保和. 加参强心方治疗冠心病心力衰竭气虚血瘀证疗效观察[J]. 河南中医,2007,27(7):23-24. DOI:10.3969/j.issn.1003-5028.2007.07.011.
51. 周桦. 葶苈生脉方对心衰大鼠AngⅡ及其受体信号传导的影响[D]. 河北:河北医科大学,2009. DOI:10.7666/d.y1637601.
52. 姜冬云. 温心颗粒治疗慢性充血性心力衰竭作用机理研究[D].成都中医药大学,2008.
53. 邹旭,牛平平,姚耿圳,等. 暖心胶囊对心衰大鼠肌浆网Ca2+-ATP酶、血浆AngⅡ、ALD以及血流动力学的影响[J]. 新中医,2011,43(11):110-112.

**Participant:**

1. 闵红燕,江柳,张密浓.心宝丸对老年慢性心功能不全患者运动耐量和B型脑钠肽前体水平的影响[J].中国实用医药,2018,13(32):16-18.
2. 于凯成.心力衰竭的中医药治疗[J].中国社区医师,1996(12):21-25.
3. 赵宇程,陆备军,庄亚杰,等. 硝苯地平加心宝丸治疗缓慢性心律失常的临床分析[J]. 浙江临床医学,2009,11(1):73-74. DOI:10.3969/j.issn.1008-7664.2009.01.039.

**Awaiting classification:**

1. 陈勇鹏. 比索洛尔联合心宝丸治疗冠心病心力衰竭效果观察[J]. 基层医学论坛,2012,16(19):2516-2517. DOI:10.3969/j.issn.1672-1721.2012.19.049.
2. 陈勇鹏.卡维地洛联合曲美他嗪 心宝丸治疗慢性心力衰竭疗效观察[J].基层医学论坛,2013(1):4-6.
3. 陈振岭,郑玉水,李为,等. 厄贝沙坦联合心宝丸治疗老年慢性心力衰竭合并窦性心动过缓的临床效果[J]. 中国医药导报,2018,15(2):54-57.
4. 高冶,王学磊,薛敏. 心宝丸治疗合并心动过缓的慢性心力衰竭的疗效观察[J]. 内蒙古医学杂志,2016,48(4):471-472. DOI:10.16096/J.cnki.nmgyxzz.2016.48.04.034.
5. 李静,吕国芬. 心宝丸联合美托洛尔治疗慢性心力衰竭的临床研究[J]. 现代药物与临床,2019,34(5):1294-1297. DOI:10.7501/j.issn.1674-5515.2019.05.006.
6. 李秋君.心宝丸联合曲美他嗪治疗慢性心衰合并窦性心动过缓的临床效果评价[J].中西医结合心血管病电子杂志,2019,7(27):4-5.
7. 刘林强,王玲,孙雷雷. 心宝丸治疗终末期心力衰竭的疗效观察[J]. 中国实用乡村医生杂志,2015(5):55-56. DOI:10.3969/j.issn.1672-7185.2015.05.033.
8. 卢方贵,徐义,黄素素.厄贝沙坦联合心宝丸治疗老年慢性心力衰竭合并窦性心动过缓的临床效果分析[J].北方药学,2020,17(6):116-117.
9. 王彪,尤冬霞,赵林凤.厄贝沙坦联合心宝丸治疗老年慢性心力衰竭合并窦性心动过缓的效果[J].中西医结合心血管病电子杂志,2018,6(22):113.
10. 王治国,郑艳妮,沙海旺. 心宝丸联合米力农治疗终末期心力衰竭的临床研究[J]. 现代药物与临床,2019,34(9):2603-2608. DOI:10.7501/j.issn.1674-5515.2019.09.006.
11. 魏勇,周志奇,邵静. 心宝丸治疗慢性收缩性心力衰竭60例临床观察[J]. 中医临床研究,2014(13):63-64. DOI:10.3969/j.issn.1674-7860.2014.13.038.
12. 吴继雷,冯秀芝,王凤荣. 心宝丸治疗心肌梗死PCI术后合并慢性心力衰竭的疗效观察[J]. 中国实用医药,2020,15(16):104-106. DOI:10.14163/j.cnki.11-5547/r.2020.16.044.
13. 徐静,齐惠英. 心宝丸联合卡维地洛治疗慢性心力衰竭的临床研究[J]. 现代药物与临床,2019,34(12):3539-3542. DOI:10.7501/j.issn.1674-5515.2019.12.008.
14. 张莹莹.心宝丸联合曲美他嗪治疗慢性心衰合并窦性心动过缓的临床疗效观察[J].湖北中医杂志,2016,38(4):3-5.
15. 赵雪莲,郭文娟,郑铎. 厄贝沙坦联合心宝丸治疗老年慢性心力衰竭合幵窦性心动过缓的临床效果[J]. 中国药物经济学,2019,14(1):48-51. DOI:10.12010/j.issn.1673-5846.2019.01.011.
16. 黄亚莉. 心宝丸联合比索洛尔治疗冠心病心力衰竭疗效观察[J]. 陕西中医,2014(11):1480-1481. DOI:10.3969/j.issn.1000-7369.2014.11.023.

# Supplementary File S4. A list of excluded studies by reading full text.

**Type of Outcome Measures**

1. 陈勇鹏. 比索洛尔联合心宝丸治疗冠心病心力衰竭效果观察[J]. 基层医学论坛,2012,16(19):2516-2517. DOI:10.3969/j.issn.1672-1721.2012.19.049.
2. 刘林强,王玲,孙雷雷. 心宝丸治疗终末期心力衰竭的疗效观察[J]. 中国实用乡村医生杂志,2015(5):55-56. DOI:10.3969/j.issn.1672-7185.2015.05.033.
3. 王彪,尤冬霞,赵林凤.厄贝沙坦联合心宝丸治疗老年慢性心力衰竭合并窦性心动过缓的效果[J].中西医结合心血管病电子杂志,2018,6(22):113.
4. 黄亚莉. 心宝丸联合比索洛尔治疗冠心病心力衰竭疗效观察[J]. 陕西中医,2014(11):1480-1481. DOI:10.3969/j.issn.1000-7369.2014.11.023.
5. 吴继雷,冯秀芝,王凤荣. 心宝丸治疗心肌梗死PCI术后合并慢性心力衰竭的疗效观察[J]. 中国实用医药,2020,15(16):104-106. DOI:10.14163/j.cnki.11-5547/r.2020.16.044.
6. 魏勇,周志奇,邵静. 心宝丸治疗慢性收缩性心力衰竭60例临床观察[J]. 中医临床研究,2014(13):63-64. DOI:10.3969/j.issn.1674-7860.2014.13.038.

**Intervention:**

陈勇鹏.卡维地洛联合曲美他嗪心宝丸治疗慢性心力衰竭疗效观察[J].基层医学论坛,2013(1):4-6.

# Supplementary File S5. Summary table of the studies include.

| **Study** | **Formulation** | **Source** | **Species, concentration** | **Quality control reported**  **(Y/N)** | **Chemical analysis reported**  **(Y/N)** |
| --- | --- | --- | --- | --- | --- |
| Chen ZL  2018 | Xinbao pill | Guangdong Xinbao Pharmaceutical Technology Co., Ltd. (Guangzhou, China) | - Moschus (the dried preputial secretion of Moschus berezovskii, M. sifanicus or M. moschiferus),   concentration uncertainty   - Panax quinquefolius L. [Araliaceae], Dried roots and rhizomes of Panax ginseng C. A. Mey,   concentration uncertainty   - Cinnamomum verum J.Presl [Lauraceae], Dried bark of Cinnamomum cassia Presl,   concentration uncertainty   - Datura metel L. [Solanaceae], Dry flowers of Datura metel L.   concentration uncertainty   - Aconitum carmichaeli Debeaux [Ranunculaceae],Processed product of the sub-root of Aconitum carmichaelii Debx,   concentration uncertainty   - Panax notoginseng (Burkill) F.H.Chen [Araliaceae], Dried roots and rhizomes of Panax notoginseng (Burk.) F. H. Chen,   concentration uncertainty   - Bufonis Venenum (the dry secretion of Bufo bufo gargarizans Cantor or Bufo melanostictus Schneider),   concentration uncertainty   - Cervi Cornu Pantotrichum (the unossitized, densely hairy young horn of a buck by Cervus Nippon Temminck or Cervus elaphus Linnaeus),   concentration uncertainty   - Borneolum Syntheticum.   concentration uncertainty | Y – Prepared according to the Pharmacopoeia of China, 2010 edition | N |
| Gao Y  2016 | Xinbao pill | Guangdong Xinbao Pharmaceutical Technology Co., Ltd. (Guangzhou, China) | - Moschus (the dried preputial secretion of Moschus berezovskii, M. sifanicus or M. moschiferus),   concentration uncertainty   - Panax quinquefolius L. [Araliaceae], Dried roots and rhizomes of Panax ginseng C. A. Mey,   concentration uncertainty   - Cinnamomum verum J.Presl [Lauraceae], Dried bark of Cinnamomum cassia Presl,   concentration uncertainty   - Datura metel L. [Solanaceae], Dry flowers of Datura metel L.   concentration uncertainty   - Aconitum carmichaeli Debeaux [Ranunculaceae],Processed product of the sub-root of Aconitum carmichaelii Debx,   concentration uncertainty   - Panax notoginseng (Burkill) F.H.Chen [Araliaceae], Dried roots and rhizomes of Panax notoginseng (Burk.) F. H. Chen,   concentration uncertainty   - Bufonis Venenum (the dry secretion of Bufo bufo gargarizans Cantor or Bufo melanostictus Schneider),   concentration uncertainty   - Cervi Cornu Pantotrichum (the unossitized, densely hairy young horn of a buck by Cervus Nippon Temminck or Cervus elaphus Linnaeus),   concentration uncertainty   - Borneolum Syntheticum.   concentration uncertainty | Y – Prepared according to the Pharmacopoeia of China, 2010 edition | N |
| Li J  2019 | Xinbao pill | Guangdong Taiantang Pharmaceutical Co., Ltd. (Shantou, China) | - Moschus (the dried preputial secretion of Moschus berezovskii, M. sifanicus or M. moschiferus),   concentration uncertainty   - Panax quinquefolius L. [Araliaceae], Dried roots and rhizomes of Panax ginseng C. A. Mey,   concentration uncertainty   - Cinnamomum verum J.Presl [Lauraceae], Dried bark of Cinnamomum cassia Presl,   concentration uncertainty   - Datura metel L. [Solanaceae], Dry flowers of Datura metel L.   concentration uncertainty   - Aconitum carmichaeli Debeaux [Ranunculaceae],Processed product of the sub-root of Aconitum carmichaelii Debx,   concentration uncertainty   - Panax notoginseng (Burkill) F.H.Chen [Araliaceae], Dried roots and rhizomes of Panax notoginseng (Burk.) F. H. Chen,   concentration uncertainty   - Bufonis Venenum (the dry secretion of Bufo bufo gargarizans Cantor or Bufo melanostictus Schneider),   concentration uncertainty   - Cervi Cornu Pantotrichum (the unossitized, densely hairy young horn of a buck by Cervus Nippon Temminck or Cervus elaphus Linnaeus),   concentration uncertainty   - Borneolum Syntheticum.   concentration uncertainty | Y – Prepared according to the Pharmacopoeia of China, 2010 edition | N |
| Li QJ  2019 | Xinbao pill | Guangdong Xinbao Pharmaceutical Technology Co., Ltd. (Guangzhou, China) | - Moschus (the dried preputial secretion of Moschus berezovskii, M. sifanicus or M. moschiferus),   concentration uncertainty   - Panax quinquefolius L. [Araliaceae], Dried roots and rhizomes of Panax ginseng C. A. Mey,   concentration uncertainty   - Cinnamomum verum J.Presl [Lauraceae], Dried bark of Cinnamomum cassia Presl,   concentration uncertainty   - Datura metel L. [Solanaceae], Dry flowers of Datura metel L.   concentration uncertainty   - Aconitum carmichaeli Debeaux [Ranunculaceae],Processed product of the sub-root of Aconitum carmichaelii Debx,   concentration uncertainty   - Panax notoginseng (Burkill) F.H.Chen [Araliaceae], Dried roots and rhizomes of Panax notoginseng (Burk.) F. H. Chen,   concentration uncertainty   - Bufonis Venenum (the dry secretion of Bufo bufo gargarizans Cantor or Bufo melanostictus Schneider),   concentration uncertainty   - Cervi Cornu Pantotrichum (the unossitized, densely hairy young horn of a buck by Cervus Nippon Temminck or Cervus elaphus Linnaeus),   concentration uncertainty   - Borneolum Syntheticum.   concentration uncertainty | Y – Prepared according to the Pharmacopoeia of China, 2010 edition | N |
| Lu FG  2020 | Xinbao pill | Beijing Tongrentang Co., Ltd. (Beijing, China) | - Moschus (the dried preputial secretion of Moschus berezovskii, M. sifanicus or M. moschiferus),   concentration uncertainty   - Panax quinquefolius L. [Araliaceae], Dried roots and rhizomes of Panax ginseng C. A. Mey,   concentration uncertainty   - Cinnamomum verum J.Presl [Lauraceae], Dried bark of Cinnamomum cassia Presl,   concentration uncertainty   - Datura metel L. [Solanaceae], Dry flowers of Datura metel L.   concentration uncertainty   - Aconitum carmichaeli Debeaux [Ranunculaceae],Processed product of the sub-root of Aconitum carmichaelii Debx,   concentration uncertainty   - Panax notoginseng (Burkill) F.H.Chen [Araliaceae], Dried roots and rhizomes of Panax notoginseng (Burk.) F. H. Chen,   concentration uncertainty   - Bufonis Venenum (the dry secretion of Bufo bufo gargarizans Cantor or Bufo melanostictus Schneider),   concentration uncertainty   - Cervi Cornu Pantotrichum (the unossitized, densely hairy young horn of a buck by Cervus Nippon Temminck or Cervus elaphus Linnaeus),   concentration uncertainty   - Borneolum Syntheticum.   concentration uncertainty | Y – Prepared according to the Pharmacopoeia of China, 2010 edition | N |
| Wang ZG  2019 | Xinbao pill | Guangdong Xinbao Pharmaceutical Technology Co., Ltd. (Guangzhou, China) | - Moschus (the dried preputial secretion of Moschus berezovskii, M. sifanicus or M. moschiferus),   concentration uncertainty   - Panax quinquefolius L. [Araliaceae], Dried roots and rhizomes of Panax ginseng C. A. Mey,   concentration uncertainty   - Cinnamomum verum J.Presl [Lauraceae], Dried bark of Cinnamomum cassia Presl,   concentration uncertainty   - Datura metel L. [Solanaceae], Dry flowers of Datura metel L.   concentration uncertainty   - Aconitum carmichaeli Debeaux [Ranunculaceae],Processed product of the sub-root of Aconitum carmichaelii Debx,   concentration uncertainty   - Panax notoginseng (Burkill) F.H.Chen [Araliaceae], Dried roots and rhizomes of Panax notoginseng (Burk.) F. H. Chen,   concentration uncertainty   - Bufonis Venenum (the dry secretion of Bufo bufo gargarizans Cantor or Bufo melanostictus Schneider),   concentration uncertainty   - Cervi Cornu Pantotrichum (the unossitized, densely hairy young horn of a buck by Cervus Nippon Temminck or Cervus elaphus Linnaeus),   concentration uncertainty   - Borneolum Syntheticum.   concentration uncertainty | Y – Prepared according to the Pharmacopoeia of China, 2010 edition | N |
| Xu J  2019 | Xinbao pill | Guangdong Taiantang Pharmaceutical Co., Ltd. (Shantou, China) | - Moschus (the dried preputial secretion of Moschus berezovskii, M. sifanicus or M. moschiferus),   concentration uncertainty   - Panax quinquefolius L. [Araliaceae], Dried roots and rhizomes of Panax ginseng C. A. Mey,   concentration uncertainty   - Cinnamomum verum J.Presl [Lauraceae], Dried bark of Cinnamomum cassia Presl,   concentration uncertainty   - Datura metel L. [Solanaceae], Dry flowers of Datura metel L.   concentration uncertainty   - Aconitum carmichaeli Debeaux [Ranunculaceae],Processed product of the sub-root of Aconitum carmichaelii Debx,   concentration uncertainty   - Panax notoginseng (Burkill) F.H.Chen [Araliaceae], Dried roots and rhizomes of Panax notoginseng (Burk.) F. H. Chen,   concentration uncertainty   - Bufonis Venenum (the dry secretion of Bufo bufo gargarizans Cantor or Bufo melanostictus Schneider),   concentration uncertainty   - Cervi Cornu Pantotrichum (the unossitized, densely hairy young horn of a buck by Cervus Nippon Temminck or Cervus elaphus Linnaeus),   concentration uncertainty   - Borneolum Syntheticum.   concentration uncertainty | Y – Prepared according to the Pharmacopoeia of China, 2010 edition | N |
| Zhang YY 2016 | Xinbao pill | Guangdong Xinbao Pharmaceutical Technology Co., Ltd. (Guangzhou, China) | - Moschus (the dried preputial secretion of Moschus berezovskii, M. sifanicus or M. moschiferus),   concentration uncertainty   - Panax quinquefolius L. [Araliaceae], Dried roots and rhizomes of Panax ginseng C. A. Mey,   concentration uncertainty   - Cinnamomum verum J.Presl [Lauraceae], Dried bark of Cinnamomum cassia Presl,   concentration uncertainty   - Datura metel L. [Solanaceae], Dry flowers of Datura metel L.   concentration uncertainty   - Aconitum carmichaeli Debeaux [Ranunculaceae],Processed product of the sub-root of Aconitum carmichaelii Debx,   concentration uncertainty   - Panax notoginseng (Burkill) F.H.Chen [Araliaceae], Dried roots and rhizomes of Panax notoginseng (Burk.) F. H. Chen,   concentration uncertainty   - Bufonis Venenum (the dry secretion of Bufo bufo gargarizans Cantor or Bufo melanostictus Schneider),   concentration uncertainty   - Cervi Cornu Pantotrichum (the unossitized, densely hairy young horn of a buck by Cervus Nippon Temminck or Cervus elaphus Linnaeus),   concentration uncertainty   - Borneolum Syntheticum.   concentration uncertainty | Y – Prepared according to the Pharmacopoeia of China, 2010 edition | N |
| Zhao XL  2019 | Xinbao pill | Guangdong Xinbao Pharmaceutical Technology Co., Ltd. (Guangzhou, China) | - Moschus (the dried preputial secretion of Moschus berezovskii, M. sifanicus or M. moschiferus),   concentration uncertainty   - Panax quinquefolius L. [Araliaceae], Dried roots and rhizomes of Panax ginseng C. A. Mey,   concentration uncertainty   - Cinnamomum verum J.Presl [Lauraceae], Dried bark of Cinnamomum cassia Presl,   concentration uncertainty   - Datura metel L. [Solanaceae], Dry flowers of Datura metel L.   concentration uncertainty   - Aconitum carmichaeli Debeaux [Ranunculaceae],Processed product of the sub-root of Aconitum carmichaelii Debx,   concentration uncertainty   - Panax notoginseng (Burkill) F.H.Chen [Araliaceae], Dried roots and rhizomes of Panax notoginseng (Burk.) F. H. Chen,   concentration uncertainty   - Bufonis Venenum (the dry secretion of Bufo bufo gargarizans Cantor or Bufo melanostictus Schneider),   concentration uncertainty   - Cervi Cornu Pantotrichum (the unossitized, densely hairy young horn of a buck by Cervus Nippon Temminck or Cervus elaphus Linnaeus),   concentration uncertainty   - Borneolum Syntheticum.   concentration uncertainty | Y – Prepared according to the Pharmacopoeia of China, 2010 edition | N |

# Supplementary File S6. Results of subgroup analysis

## 6.1 Subgroup analysis of the LVEF according to the XBP does.


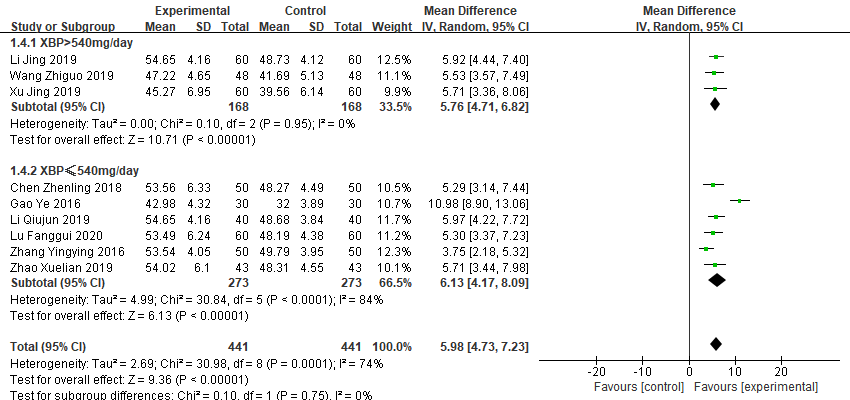


## 6.2 Subgroup analysis of the LVEF according to the treatment duration.

##
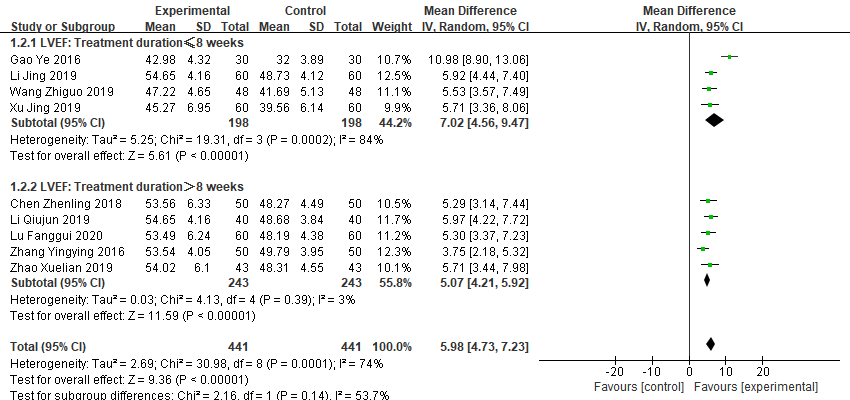


## 6.3 Subgroup analysis of the LVEF according to XBP combined with different conventional medicines.


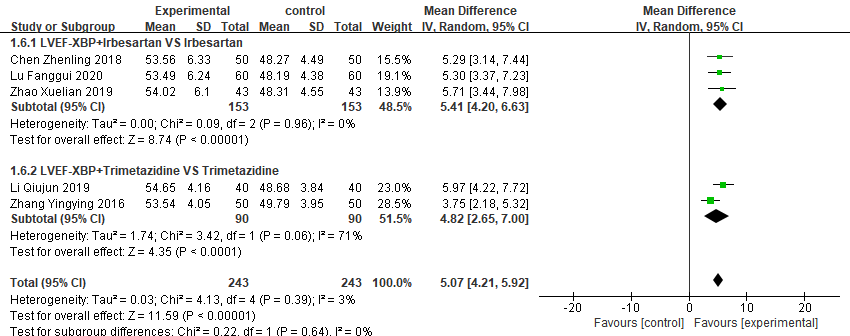


## 6.4 Subgroup analysis of the total effective rate according to the XBP does.


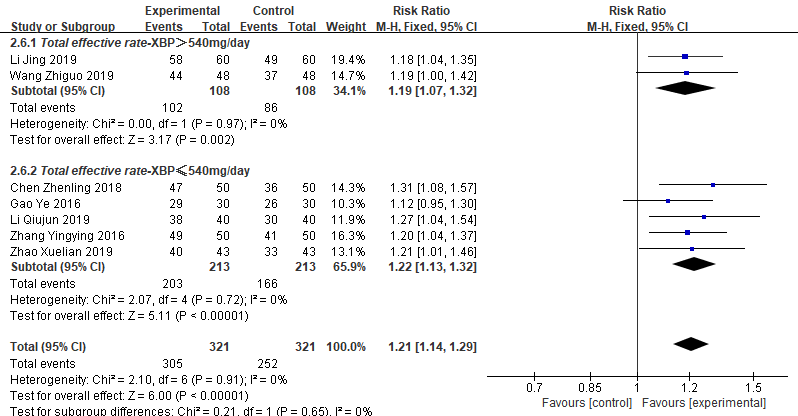


## 6.5 Subgroup analysis of the total effective rate according to the treatment duration.


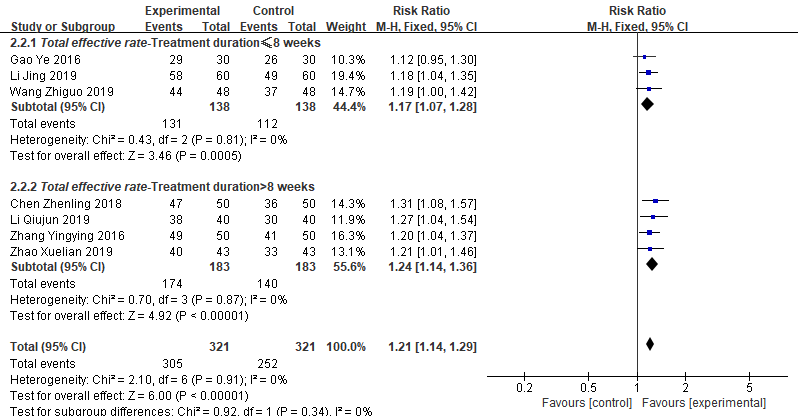


## 6.6 Subgroup analysis of the total effective rate according to XBP combined with different conventional medicines.

##
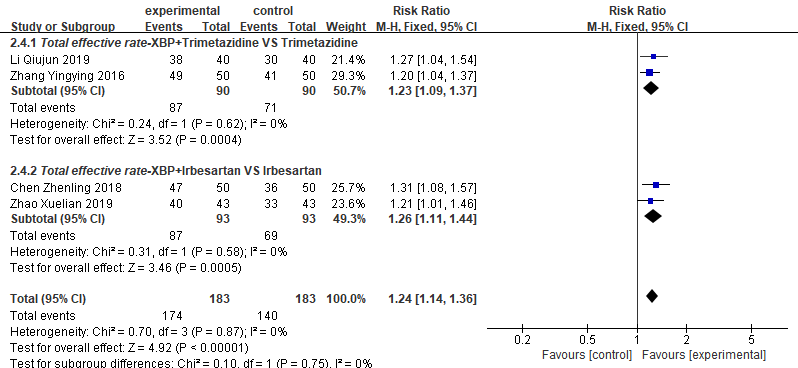


## 6.7 Subgroup analysis of LVEDD according to the XBP does.


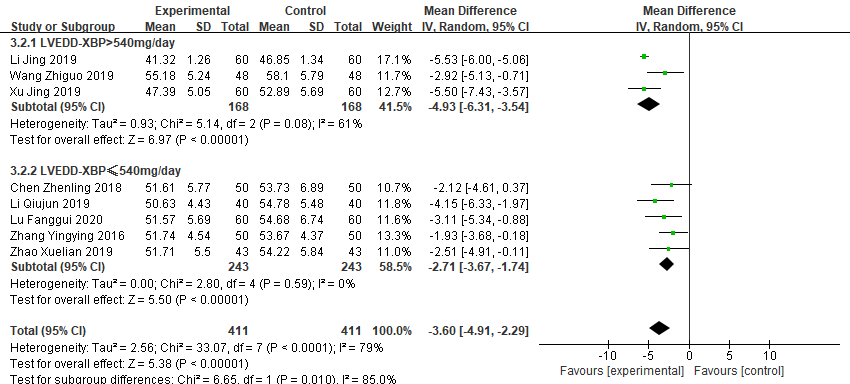


## 6.8 Subgroup analysis of LVEDD according to the treatment duration.


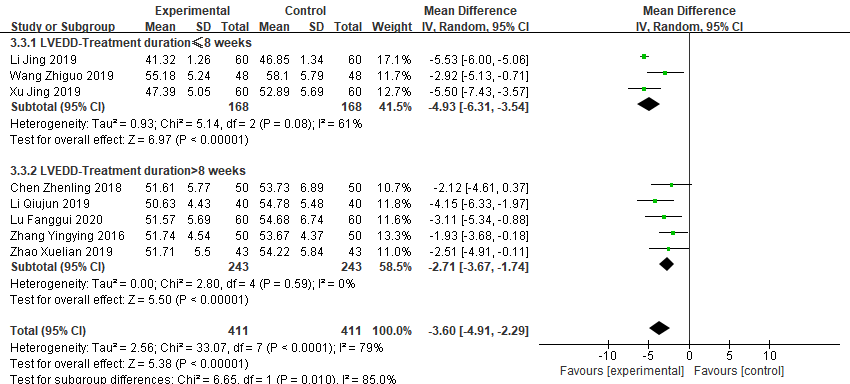


## 6.9 Subgroup analysis of LVEDD according to XBP combined with different conventional medicines.


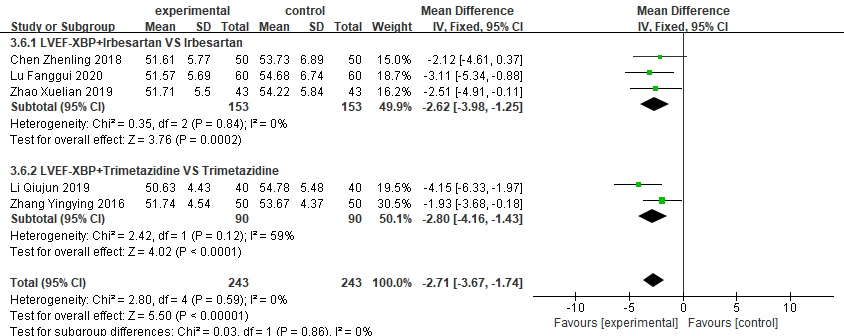


## 6.10 Subgroup analysis of LVESD according to the XBP does.


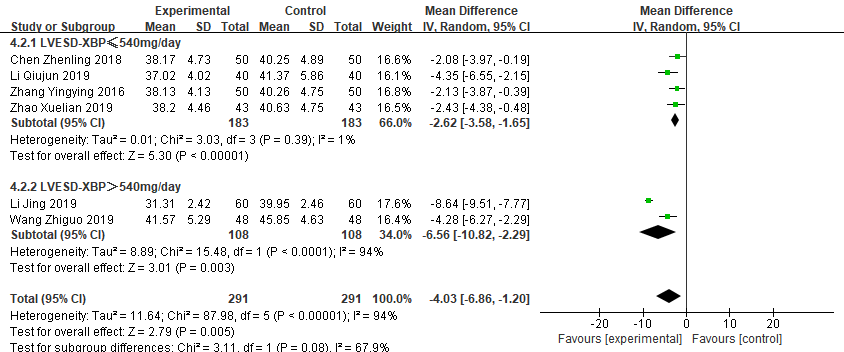


## 6.11 Subgroup analysis of LVESD according to the treatment duration.


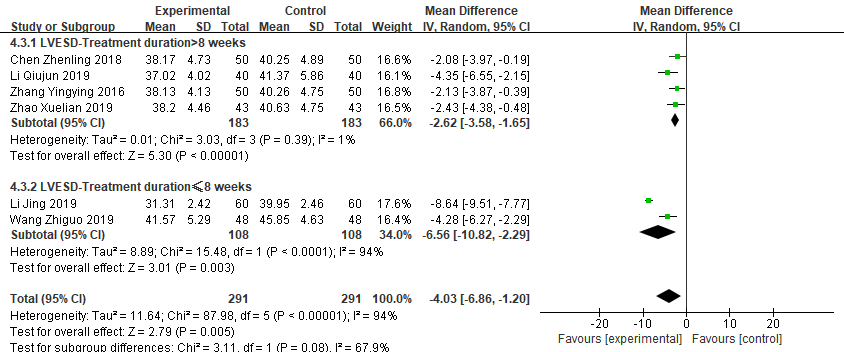


## 6.12 Subgroup analysis of LVESD according to XBP combined with different conventional medicines.


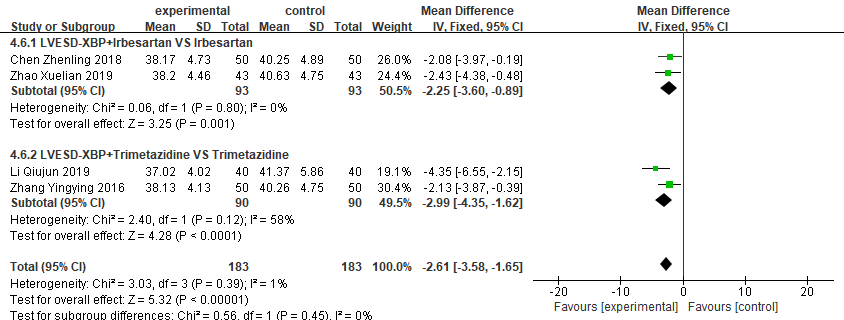


## 6.13 Subgroup analysis of 6-MWD according to the XBP does.


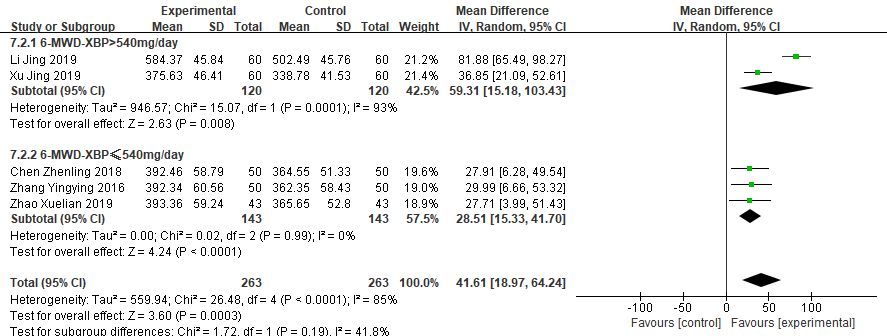


## 6.14 Subgroup analysis of 6-MWD according to the treatment duration.

##
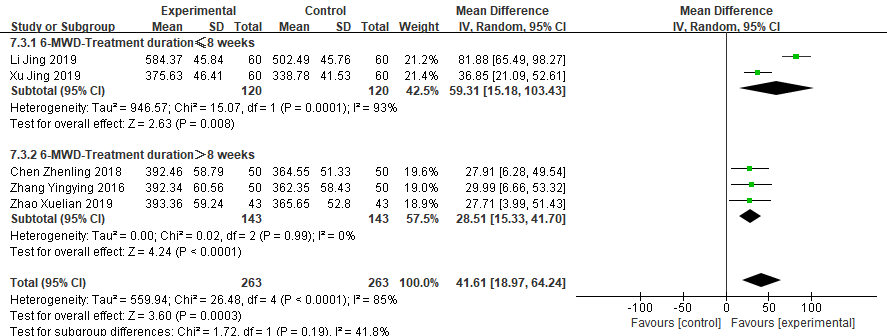


# Supplementary File S7. Results of meta-analysis and quality of evidence

| **Outcomes** | **Subgroup** | **No. participants (studies)** | **Anticipated absolute effects (95% CI)** | | **Relative effect (95% CI)** | ***I^2^* value** | **Quality of evidence (GRADE)** | **Comments** |
| --- | --- | --- | --- | --- | --- | --- | --- | --- |
|  |  |  | **Risk with control group** | **Risk with intervention group** |  |  |  |  |
|  |  |  |  |  |  |  |  |  |
| Subgroup 1  (Treatment duration) | ≤8 weeks | 396 (4 studies) | - | MD 7.02 higher (4.56 higher to 9.47 higher) | - | 84 | ⨁◯◯◯ VERY LOW | Risk of bias (-1)^a^ Inconsistency (-2)^b^ |
|  | ＞8 weeks | 486 (5 studies) | - | MD 5.07 higher (4.21 higher to 5.92 higher) | - | 3 | ⨁⨁⨁◯ MODERATE | Risk of bias (-1)^a^ |
| Subgroup 2  (XBP does) | ＞540mg/d | 336 (3 studies) | - | MD 5.76 higher (4.71 higher to 6.82 higher) | - | 0 | ⨁⨁⨁◯ MODERATE | Risk of bias (-1)^a^ |
|  | ≤540mg/d | 546 (6 studies) | - | MD 6.13 higher (4.17 higher to 8.09 higher) | - | 84 | ⨁◯◯◯ VERY LOW | Risk of bias (-1)^a^ Inconsistency (-2)^b^ |
| Subgroup 3  (XBP plus different conventional medicines) | Irbesartan | 306 (3 studies) | - | MD 5.41 higher (4.2 higher to 6.63 higher) | - | 0 | ⨁⨁⨁◯ MODERATE | Risk of bias (-1)^a^ |
|  | Trimetazidine | 180 (2 studies) | - | MD 4.82 higher (2.65 higher to 7 higher) | - | 3 | ⨁⨁◯◯  LOW | Risk of bias (-1)^a^  Imprecision(-1)^c^ |
| Total effective rate | Total | 642 (7 studies) | 785 per 1,000 | 950 per 1,000 (895 to 1,000) | RR 1.21 (1.14 to 1.29) | 0 | ⨁⨁⨁◯ MODERATE | Risk of bias (-1)^a^ |
| Subgroup 1  (Treatment duration) | ≤8 weeks | 276 (3 studies) | 812 per 1,000 | 950 per 1,000 (868 to 1,000) | RR 1.17 (1.07 to 1.28) | 0 | ⨁⨁⨁◯ MODERATE | Risk of bias (-1)^a^ |
|  | ＞8 weeks | 366 (4 studies) | 765 per 1,000 | 949 per 1,000 (872 to 1,000) | RR 1.24 (1.14 to 1.36) | 0 | ⨁⨁⨁◯ MODERATE | Risk of bias (-1)^a^ |
| Subgroup 2  (XBP does) | ＞540mg/d | 216 (2 studies) | 796 per 1,000 | 948 per 1,000 (852 to 1,000) | RR 1.19 (1.07 to 1.32) | 0 | ⨁⨁⨁◯ MODERATE | Risk of bias (-1)^a^ |
|  | ≤540mg/d | 426 (5 studies) | 779 per 1,000 | 951 per 1,000 (881 to 1,000) | RR 1.22 (1.13 to 1.32) | 0 | ⨁⨁⨁◯ MODERATE | Risk of bias (-1)^a^ |
| Subgroup 3  (XBP plus different conventional medicines) | Irbesartan | 186 (2 studies) | 742 per 1,000 | 935 per 1,000 (824 to 1,000) | RR 1.26 (1.11 to 1.44) | 0 | ⨁⨁⨁◯ MODERATE | Risk of bias (-1)^a^ |
|  | Trimetazidine | 180 (2 studies) | 789 per 1,000 | 970 per 1,000 (860 to 1,000) | RR 1.23 (1.09 to 1.37) | 0 | ⨁⨁⨁◯ MODERATE | Risk of bias (-1)^a^ |
| LVEDD | Total | 702 (7 studies) | - | MD 3.22 lower (4.03 lower to 2.42 lower) | - | 36 | ⨁⨁⨁◯ MODERATE | Risk of bias (-1)^a^ |
| Subgroup 1  (Treatment duration) | ≤8 weeks | 336 (3 studies) | - | MD 4.93 lower (6.31 lower to 3.54 lower) | - | 61 | ⨁⨁◯◯  LOW | Risk of bias (-1)^a^  Inconsistency (-1)^d^ |
|  | ＞8 weeks | 486 (5 studies) | - | MD 2.71 lower (3.67 lower to 1.74 lower) | - | 0 | ⨁⨁⨁◯ MODERATE | Risk of bias (-1)^a^ |
| Subgroup 2  (XBP does) | ＞540mg/d | 336 (3 studies) | - | MD 4.93 lower (6.31 lower to 3.54 lower) | - | 61 | ⨁⨁◯◯  LOW | Risk of bias (-1)^a^  Inconsistency (-1)^d^ |
|  | ≤540mg/d | 486 (5 studies) | - | MD 2.71 lower (3.67 lower to 1.74 lower) | - | 0 | ⨁⨁⨁◯ MODERATE | Risk of bias (-1)^a^ |
| Subgroup 3  (XBP plus different conventional medicines) | Irbesartan | 306 (3 studies) | - | MD 2.62 lower (3.98 lower to 1.25 lower) | - | 0 | ⨁⨁⨁◯ MODERATE | Risk of bias (-1)^a^ |
|  | Trimetazidine | 180 (2 studies) | - | MD 2.8 lower (4.16 lower to 1.43 lower) | - | 59 | ⨁◯◯◯ VERY LOW | Risk of bias (-1)^a^  Imprecision(-1)^c^  Inconsistency (-1)^d^ |
| LVESD | Total | 462 (5 studies) | - | MD 2.93 lower (3.8 lower to 2.06 lower) | - | 23 | ⨁⨁⨁◯ MODERATE | Risk of bias (-1)^a^ |
| Subgroup 1  (Treatment duration) | ≤8 weeks | 216 (2 studies) | - | MD 6.56 lower (10.82 lower to 2.29 lower) | - | 94 | ⨁◯◯◯ VERY LOW | Risk of bias (-1)^a^ Inconsistency (-2)^b^ |
|  | ＞8 weeks | 366 (4 studies) | - | MD 2.62 lower (3.58 lower to 1.65 lower) | - | 1 | ⨁⨁⨁◯ MODERATE | Risk of bias (-1)^a^ |
| Subgroup 2  (XBP does) | ＞540mg/d | 216 (2 studies) | - | MD 6.56 lower (10.82 lower to 2.29 lower) | - | 94 | ⨁◯◯◯ VERY LOW | Risk of bias (-1)^a^ Inconsistency (-2)^b^ |
|  | ≤540mg/d | 366 (4 studies) | - | MD 2.62 lower (3.58 lower to 1.65 lower) | - | 1 | ⨁⨁⨁◯ MODERATE | Risk of bias (-1)^a^ |
| Subgroup 3  (XBP plus different conventional medicines) | Irbesartan | 186 (2 studies) | - | MD 2.25 lower (3.6 lower to 0.89 lower) | - | 0 | ⨁⨁◯◯  LOW | Risk of bias (-1)^a^  Imprecision(-1)^c^ |
|  | Trimetazidine | 180 (2 studies) | - | MD 2.99 lower (4.35 lower to 1.62 lower) | - | 1 | ⨁⨁◯◯  LOW | Risk of bias (-1)^a^  Imprecision(-1)^c^ |
| Cardiac output | - | 320 (3 studies) | - | MD 0.56 higher (0.42 higher to 0.7 higher) | - | 0 | ⨁⨁⨁◯ MODERATE | Risk of bias (-1)^a^ |
| Stroke volume | - | 286 (3 studies) | - | MD 3.42 higher (2.03 higher to 4.81 higher) | - | 0 | ⨁⨁⨁◯ MODERATE | Risk of bias (-1)^a^ |
| 6-MWD | Total | 406 (4 studies) | - | MD 31.95 higher (21.83 higher to 42.06 higher) | - | 0 | ⨁⨁⨁◯ MODERATE | Risk of bias (-1)^a^ |
| Subgroup 1  (Treatment duration) | ≤8 weeks | 240 (2 studies) | - | MD 59.31 higher (15.18 higher to 103.43 higher) | - | 93 | ⨁◯◯◯ VERY LOW | Risk of bias (-1)^a^ Inconsistency (-2)^b^ |
|  | ＞8 weeks | 286 (3 studies) | - | MD 28.51 higher (15.33 higher to 41.7 higher) | - | 0 | ⨁⨁⨁◯ MODERATE | Risk of bias (-1)^a^ |
| Subgroup 2  (XBP does) | ＞540mg/d | 240 (2 studies) | - | MD 59.31 higher (15.18 higher to 103.43 higher) | - | 93 | ⨁◯◯◯ VERY LOW | Risk of bias (-1)^a^ Inconsistency (-2)^b^ |
|  | ≤540mg/d | 286 (3 studies) | - | MD 28.51 higher (15.33 higher to 41.7 higher) | - | 0 | ⨁⨁⨁◯ MODERATE | Risk of bias (-1)^a^ |

**Abbreviations.** XBP, Xinbao pill; CI, confidence interval, MD, mean difference, RR, relative risk; LVEF, left ventricular ejection fraction, LVESD, left ventricular end-systolic diameter, LVEDD, left ventricular end-diastolic dimension, 6-MWD, six-minutes walking distance, RCTs, randomized controlled trials,

a. Most information is from studies at low or unclear risk of bias,and potential limitations are likely to lower confidence in the estimate of effect.

b. The direction of the effect is different and I^2^ ≥ 75%

c. The sample size was too small

d. The direction of the effect is different and 50＜I^2^＜75%

GRADE Working Group grades of evidence
High certainty: we are very confident that the true effect lies close to that of the estimate of the effect.
Moderate certainty: we are moderately confident in the effect estimate: the true effect is likely to be close to the estimate of the effect, but there is a possibility that it is substantially different.
Low certainty: our confidence in the effect estimate is limited: the true effect may be substantially different from the estimate of the effect.
Very low certainty: we have very little confidence in the effect estimate: the true effect is likely to be substantially different from the estimate of effect.

| Supplementary File S8. AMSTAR 2 Checklist |
| --- |
| \|  \| \| --- \| \| **1. Did the research questions and inclusion criteria for the review include the components of PICO?** \| \| \| For Yes: \| Optional (recommended) \|  \| \| --- \| --- \| --- \| \|  Population \|  Timeframe for follow up \|  Yes   No \| \|  Intervention \|  \| \|  Comparator group \|  \| \|  Outcome \|  \| \| \|  \| |
| \| **2. Did the report of the review contain an explicit statement that the review methods were established prior to the conduct of the review and did the report justify any significant deviations from the protocol?** \| \| --- \| \| \| For Partial Yes:  The authors state that they had a written protocol or guide that included ALL the following: \| For Yes:  As for partial yes, plus the protocol should be registered and should also have specified: \|  \| \| --- \| --- \| --- \| \|  review question(s) \|  a meta-analysis/synthesis plan, if appropriate, and \|  Yes   Partial Yes   No \| \|  a search strategy \|  a plan for investigating causes of heterogeneity \| \|  inclusion/exclusion criteria \|  a plan for investigating causes of heterogeneity \| \|  a risk of bias assessment \|  \| \| |
|  |
| \| **3. Did the review authors explain their selection of the study designs for inclusion in the review?** \| \| --- \| \| \| For Yes, the review should satisfy ONE of the following: \|  \| \| --- \| --- \| \|  Explanation for including only RCTs \|  Yes   No \| \|  OR Explanation for including only NRSI \| \|  OR Explanation for including both RCTs and NRSI \| \| |
|  |
| \| **4. Did the review authors use a comprehensive literature search strategy?** \| \| --- \| \| \| For Partial Yes (all the following): \| For Yes, should also have (all the following): \|  \| \| --- \| --- \| --- \| \|  searched at least 2 databases (relevant to research question) \|  searched the reference lists / bibliographies of included studies \|  Yes   Partial Yes   No \| \|  provided key word and/or search strategy \|  searched trial/study registries \| \|  justified publication restrictions (e.g. language) \|  included/consulted content experts in the field \| \|  \|  where relevant, searched for grey literature \| \|  \|  conducted search within 24 months of completion of the review \| \| |
|  |
| \| **5. Did the review authors perform study selection in duplicate?** \| \| --- \| \| \| For Yes, either ONE of the following: \|  \| \| --- \| --- \| \|  at least two reviewers independently agreed on selection of eligible studies and achieved consensus on which studies to include \|  Yes   No \| \|  OR two reviewers selected a sample of eligible studies and achieved good agreement (at least 80 percent), with the remainder selected by one reviewer. \| \| |
|  |
| \| **6. Did the review authors perform data extraction in duplicate?** \| \| --- \| \| \| For Yes, either ONE of the following: \|  \| \| --- \| --- \| \|  at least two reviewers achieved consensus on which data to extract from included studies \|  Yes   No \| \|  OR two reviewers extracted data from a sample of eligible studies and achieved good agreement (at least 80 percent), with the remainder extracted by one reviewer. \| \| |
|  |
| \| **7. Did the review authors provide a list of excluded studies and justify the exclusions?** \| \| --- \| \| \| For Partial Yes: \| For Yes, must also have: \|  \| \| --- \| --- \| --- \| \| provided a list of all potentially relevant studies that were read in full-text form but excluded from the review \| Justified the exclusion from the review of each potentially relevant study \|  Yes   Partial Yes   No \| \| |
|  |
| \| **8. Did the review authors describe the included studies in adequate detail?** \| \| --- \| \| \| For Partial Yes (ALL the following): \| For Yes, should also have ALL the following: \|  \| \| --- \| --- \| --- \| \|  described populations \|  described population in detail \|  Yes   Partial Yes   No \| \|  described interventions \|  described intervention in detail (including doses where relevant) \| \|  described comparators \|  described comparator in detail (including doses where relevant) \| \|  described outcomes \|  described study’s setting \| \|  described research designs \|  timeframe for follow-up \| \| |
|  |
| \| **9. Did the review authors use a satisfactory technique for assessing the risk of bias (RoB) in individual studies that were included in the review?** \| \| --- \| \| \| **RCTs** \|  \|  \| \| --- \| --- \| --- \| \| For Partial Yes, must have assessed RoB from \| For Yes, must also have assessed RoB from: \|  \| \|  unconcealed allocation, and \|  allocation sequence that was not truly random, and \|  Yes   Partial Yes   No   Includes only NRSI \| \|  lack of blinding of patients and assessors when assessing outcomes (unnecessary for objective outcomes such as all-cause mortality) \|  selection of the reported result from among multiple measurements or analyses of a specified outcome \| \| **NRSI** \|  \|  \| \| For Partial Yes, must have assessed RoB: \| For Yes, must also have assessed RoB: \|  \| \|  from confounding, and \|  methods used to ascertain exposures and outcomes, and \|  Yes   Partial Yes   No   Includes only RCTs \| \|  from selection bias \|  selection of the reported result from among multiple measurements or analyses of a specified outcome \| \| |
|  |
| \| **10. Did the review authors report on the sources of funding for the studies included in the review?** \| \| --- \| \| \| For Yes \|  \| \| --- \| --- \| \|  Must have reported on the sources of funding for individual studies included in the review. Note: Reporting that the reviewers looked for this information but it was not reported by study authors also qualifies \|  Yes   No \| \| |
|  |
| \| **11. If meta-analysis was performed did the review authors use appropriate methods for statistical combination of results?** \| \| --- \| \| \| **RCTs** \|  \| \| --- \| --- \| \| For Yes: \|  \| \|  The authors justified combining the data in a meta-analysis \|  Yes   No   No meta-analysis conducted \| \|  AND they used an appropriate weighted technique to combine study results and adjusted for heterogeneity if present. \| \|  AND investigated the causes of any heterogeneity \| \|  \|  \| \| **For NRSI**  For Yes: \|  \| \|  The authors justified combining the data in a meta-analysis \|  Yes   No   No meta-analysis conducted \| \|  AND they used an appropriate weighted technique to combine study results, adjusting for heterogeneity if present \| \|  AND they statistically combined effect estimates from NRSI that were adjusted for confounding, rather than combining raw data, or justified combining raw data when adjusted effect estimates were not available \| \|  AND they reported separate summary estimates for RCTs and NRSI separately when both were included in the review \| \| |
|  |
| \| **12. If meta-analysis was performed, did the review authors assess the potential impact of RoB in individual studies on the results of the meta-analysis or other evidence synthesis?** \| \| --- \| \| \| For Yes: \|  \| \| --- \| --- \| \|  included only low risk of bias RCTs \|  Yes   No   No meta-analysis conducted \| \|  OR, if the pooled estimate was based on RCTs and/or NRSI at variable RoB, the authors performed analyses to investigate possible impact of RoB on summary estimates of effect. \| \| |
|  |
| \| **13. Did the review authors account for RoB in individual studies when interpreting/ discussing the results of the review?** \| \| --- \| \| \| For Yes: \|  \| \| --- \| --- \| \|  included only low risk of bias RCTs \|  Yes   No \| \|  OR, if RCTs with moderate or high RoB, or NRSI were included the review provided a discussion of the likely impact of RoB on the results \|  \| \| |
|  |
| \| **14. Did the review authors provide a satisfactory explanation for, and discussion of, any heterogeneity observed in the results of the review?** \| \| --- \| \| \| For Yes: \|  \| \| --- \| --- \| \| There was no significant heterogeneity in the results \| Yes   No \| \| OR if heterogeneity was present the authors performed an investigation of sources of any heterogeneity in the results and discussed the impact of this on the results of the review \|  \| \| |
|  |
| \| **15. If they performed quantitative synthesis did the review authors carry out an adequate investigation of publication bias (small study bias) and discuss its likely impact on the results of the review?** \| \| --- \| \| \| For Yes: \|  \| \| --- \| --- \| \| performed graphical or statistical tests for publication bias and discussed the likelihood and magnitude of impact of publication bias \| Yes   No   No meta-analysis conducted \| \| |
|  |
| \| **16. Did the review authors report any potential sources of conflict of interest, including any funding they received for conducting the review?** \| \| --- \| \| \| For Yes: \|  \| \| --- \| --- \| \| The authors reported no competing interests OR \| Yes   No \| \| The authors described their funding sources and how they managed potential conflicts of interest \| \| |
|  |
|  |
